# Shifting Mycobacterial Serine Hydrolase Activity Visualized Using Multi-Layer In-Gel Activity Assays

**DOI:** 10.3390/molecules29143386

**Published:** 2024-07-18

**Authors:** Allison L. Goss, Renee E. Shudick, R. Jeremy Johnson

**Affiliations:** Department of Chemistry and Biochemistry, Butler University, 4600 Sunset Ave., Indianapolis, IN 46208, USA

**Keywords:** esterase, serine hydrolase, fluorogenic ester, tuberculosis, *Mycobacterium tuberculosis*, diced electrophoresis gel, lipolysis

## Abstract

The ability of *Mycobacterium tuberculosis* to derive lipids from the host, store them intracellularly, and then break them down into energy requires a battery of serine hydrolases. Serine hydrolases are a large, diverse enzyme family with functional roles in dormant, active, and reactivating mycobacterial cultures. To rapidly measure substrate-dependent shifts in mycobacterial serine hydrolase activity, we combined a robust mycobacterial growth system of nitrogen limitation and variable carbon availability with nimble in-gel fluorogenic enzyme measurements. Using this methodology, we rapidly analyzed a range of ester substrates, identified multiple hydrolases concurrently, observed functional enzyme shifts, and measured global substrate preferences. Within every growth condition, mycobacterial hydrolases displayed the full, dynamic range of upregulated, downregulated, and constitutively active hydrolases independent of the ester substrate. Increasing the alkyl chain length of the ester substrate also allowed visualization of distinct hydrolase activity likely corresponding with lipases most responsible for lipid breakdown. The most robust expression of hydrolase activity was observed under the highest stress growth conditions, reflecting the induction of multiple metabolic pathways scavenging for energy to survive under this high stress. The unique hydrolases present under these high-stress conditions could represent novel drug targets for combination treatment with current front-line therapeutics. Combining diverse fluorogenic esters with in-gel activity measurements provides a rapid, customizable, and sensitive detection method for mycobacterial serine hydrolase activity.

## 1. Introduction

*Mycobacterium tuberculosis* (*Mtb*), the causative agent of tuberculosis (TB), remains one of the deadliest pathogens in the world and is responsible for the death of 1.5 to 2 million people each year [1]. *Mtb*’s success as an infectious pathogen is helped by its ability to enter a dormant state within its host [2,3]. Dormant *Mtb* is generally drug-resistant and evades immune responses, with one-quarter of the human population estimated to be infected with dormant *Mtb* [1,4]. One mechanism for dormant *Mtb* survival is to scavenge and store host cell lipids as triacylglycerols (TAGs) in intracellular lipid inclusions (ILIs) to relieve metabolic stress and provide nutrient storage [5,6,7]. These TAGs can be used as an energy source during times of stress, and their accumulation is linked to cell division arrest and drug tolerance [4,6]. Additionally, TAG breakdown is essential for reactivation to active TB as it fuels the cellular replication needed for an active infection [6,7]. *Mtb*’s ability to derive lipids from the host, store them in ILIs, and then break them down to use as nutrients for reactivation requires a battery of enzymes, including a wealth of serine hydrolases [8,9].

Serine hydrolases are a large, diverse family of enzymes with a conserved α/β hydrolase protein fold and the ability to catalyze hydrolysis reactions using a conserved catalytic triad or similar catalytic machinery [9]. Within *Mtb*, serine hydrolases are functional in dormant, active, and reactivating cultures [10,11]. Supporting their essential roles in *Mtb* metabolism, inactivation and activation patterns of serine hydrolases directly accompany the dormant to active transition in *Mtb* [5,10]. When *Mtb* is dormant, serine hydrolases are less active and fatty acids accumulate within ILIs [12]. As *Mtb* reactivates, this relationship is reversed, and lipid stores are broken down to provide *Mtb* with the energy needed for metabolism and replication [10]. Using activity-based protein profiling (ABPP) across multiple studies and growth conditions, over 70 serine hydrolases have been labeled, and their relative activities correlated with hypoxic, normoxic, reactivation, and acid stress growth conditions [10,11,13,14,15,16].

To observe the transitions from active to dormant to reactivated growth states in *Mtb*, multiple stress-dependent growth conditions have been developed to induce dormant growth and accumulation of TAG in ILIs, including nutrient starvation, hypoxia, low pH, exposure to nitric acid, long-chain fatty acid supplementation, and nitrogen limitation [5,17]. Individual growth stressors or combinatorial stressors were correlated with TAG accumulation, followed by lipid storage depletion upon return to standard growth media. One of the most robust shifts was observed using nitrogen limitation and low carbon availability (1% glycerol) with over a four-fold increase in TAG ILI storage [5]. Reactivation from nitrogen limitation has also been correlated with increased serine hydrolase activity, as two general serine hydrolase inhibitors (tetrahydrolipstatin and M*m*PPOX) block TAG hydrolysis [5]. Additionally, overexpression of LipY, a lipase hypothesized to be essential to *Mtb* TAG hydrolysis, has been shown to induce only limited shifts in TAG levels upon reactivation, indicating that a complex mixture of serine hydrolase activity is required for TAG synthesis and degradation [5]. Nitrogen limitation provides a simple, tunable mycobacterial growth system for inducing differential serine hydrolase expression and correlating this with ILI and TAG levels [5].

Our goal was to combine nitrogen limitation growth conditions with diverse fluorogenic ester substrates and in-gel enzyme activity measurements to quantitate variations in serine hydrolase activity in response to the mycobacterial dormant to active growth transition. Unlike ABPP studies, which used a single covalent serine hydrolase ligand per study [11], in-gel activity assays allow for sensitive, dynamic measurements of hydrolase activity across a range of ester substrates, reducing ligand bias and allowing the quantitation of the global substrate specificity of mycobacterial hydrolases [10,18,19]. In-gel measurements also require 50–100-fold less protein per sample than ABPP, allow rapid identification of novel reactivity, and can be used to differentiate between mycobacterial species, including active versus dormant *Mtb* [10,18,20]. Combining variable nitrogen and carbon levels with increasing reactivation periods and with a library of fluorogenic ester substrates, we determined growth-condition and substrate-dependent shifts in serine hydrolase activity. We quantitated growth-related shifts in serine hydrolase activity in response to stress levels and traced them to individual serine hydrolases. Importantly, we identified serine hydrolases upregulated under reactivation conditions that could be essential to *Mtb* persistence and survival.

## 2. Results

### 2.1. Dormant and Reactivation Growth System

To induce mycobacterial dormancy, we adapted a nitrogen-deprivation growth system for *Mycolicibacterium smegmatis* (*Msmeg*) [5]. *Msmeg* provides a model for studying nutrient transitions in mycobacteria as it undergoes the same active to dormant reactivation growth transitions while storing high quantities of TAG within ILI upon nitrogen starvation [12,21]. Most importantly, the processes requiring serine hydrolase activity, including lipid accumulation and hydrolysis, are conserved in both species, as are most of the serine hydrolase genes [11,19]. In-gel comparisons of hydrolase activity between *Mtb* and *Msmeg* also showed comparable numbers of total hydrolases and similar decreasing levels of hydrolase activity with dormancy [10,19,22].

**Figure 1:**
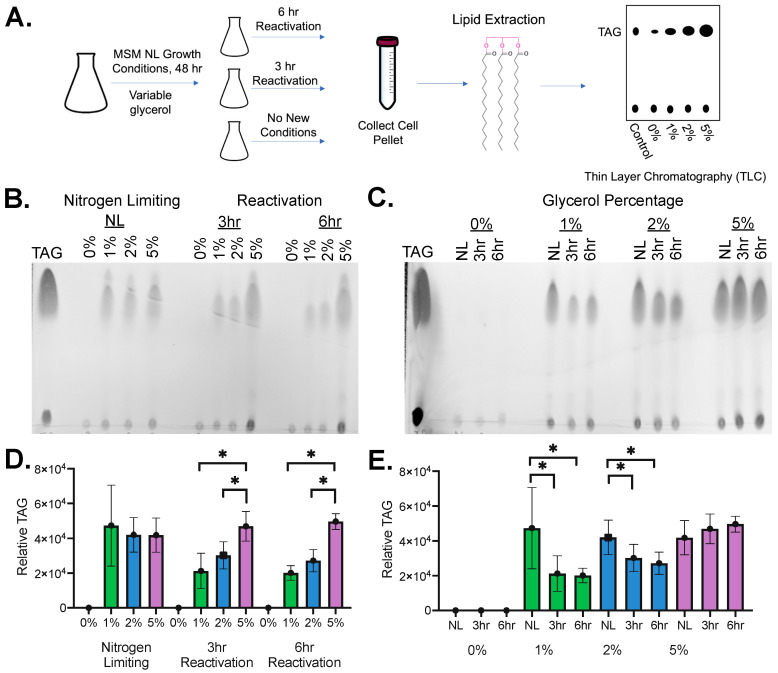
Nitrogen deprivation and carbon availability mimic the dormant and reactivation growth cycle of mycobacteria. (**A**) Growth system development and analysis. Lipids were extracted from *Msmeg* cultures and analyzed via TLC to track shifts in TAG concentration. (**B**,**C**) Extracted lipids from *Msmeg* cultures were spotted alongside TAG (triolein 18:1; Avanti). Spots were standardized to the dry weight of the mycobacterial pellet and visualized using a phosphomolybdic acid stain. (**D**,**E**) TLC analysis was quantitated using ImageJ Ver1.53 to determine the relative amount of TAG in each sample (total n = 4; 2× biological replicates, 2× technical replicates) with error bars representing a standard deviation from the mean. A 2-tailed T-test assuming unequal variance was performed; the asterisks (*) represent significant differences (*p*-value < 0.05). (**B**,**D**) Change in TAG concentration with increasing glycerol (0%, 1%, 2%, and 5%) in each growth state (NL: nitrogen limiting, 3 hr: 3 hour reactivation, 6 hr: 6 hour reactivation). (**C**,**E**) Change in TAG concentration with increasing activation time for each glycerol concentration.

After initially synchronizing all growth cultures in the Middlebrook 7H9 medium, the dormant state was induced by transitioning *Msmeg* to a nitrogen-limiting mineral salt media (MSM NL) (Figure 1A). The ability of dormant mycobacteria to accumulate lipids from a host carbon source was measured by supplementing the media with varying concentrations of glycerol (0%, 1%, 2%, and 5%). Carbon limitation led to changing levels of TAG accumulation and degradation, suggesting differential hydrolase expression. To mimic the reactivation state, 48 h dormant cultures were collected and transitioned to mineral salt media with standard nitrogen availability (MSM). Previous measurements showed that TAG consumption after reactivation was rapid within the first hours after starvation, indicating rapid changes in metabolic processes and hydrolase expression [5]. TAG consumption then continued linearly over a 24 h period in *Msmeg*, indicating steady-state levels of hydrolase expression [5]. Since we were most interested in the differential expression of hydrolases upon the transitions to dormancy and reactivation, we chose to collect reactivation samples within the first few hours post-reactivation. Specifically, we analyzed samples 3 h and 6 h post-reactivation to differentiate steady-state levels of hydrolase activity from shifting hydrolase activity.

To confirm that shifts in lipid concentrations accompanied these growth changes, lipid storage in the form of TAG was analyzed using thin-layer chromatography (TLC; Figure 1) [5]. The growth system showed a significant increase in relative TAG concentrations with increasing amounts of the supplied carbon source (1–5% glycerol) (*p*-value < 0.05) (Figure 1B,D). This mimics the mycobacterial accumulation of TAG from a host carbon source during infection [5]. This increase was not observed in the dormant (NL) samples (*p*-value > 0.05), as all three glycerol concentrations (1–5%) maintained identical levels of overall TAG (Figure 1B,D). The levels of accumulated TAG across all NL conditions were within error of the 5% glycerol samples (Figure 1B,D), likely indicating that dormant mycobacteria accumulate enough lipids to reach maximum TAG storage capacity, even with lower outside carbon availability.

Comparing TAG levels based on activation time, a significant decrease in relative TAG concentration with increasing activation time (*p*-value < 0.05) was observed, mimicking the mycobacterial breakdown of TAG required for the transition to an active infection (Figure 1C,E) [5]. The concentration of TAG in the 5% glycerol cultures, however, remained constant (*p*-value > 0.05) with increasing activation time, reflecting high lipid accumulation during dormancy and the small percentage of degradation during activation (Figure 1C,E). This matched with previous nitrogen starvation measurements where over 24 h was required to see substantial shifts in TAG levels with high carbon availability (5% glycerol) [5]. The combination of NL and no added carbon source (0% glycerol) had no measurable TAG within the sensitivity of our TLC staining, making for interesting comparison samples where hyperactive hydrolase activity may be required to maintain necessary nutrient levels for baseline growth and survival (Figure 1). With confirmed dynamic cycling of nutrient storage in our nitrogen deprivation and carbon availability growth conditions, lysates (2× biological replicates) were collected across the growth conditions and their relative shifts in serine hydrolase activity measured by in-gel enzymatic activity assays.

### 2.2. Shifted Activity of Serine Hydrolases in Dormant versus Active Growth Conditions

**Figure 2:**
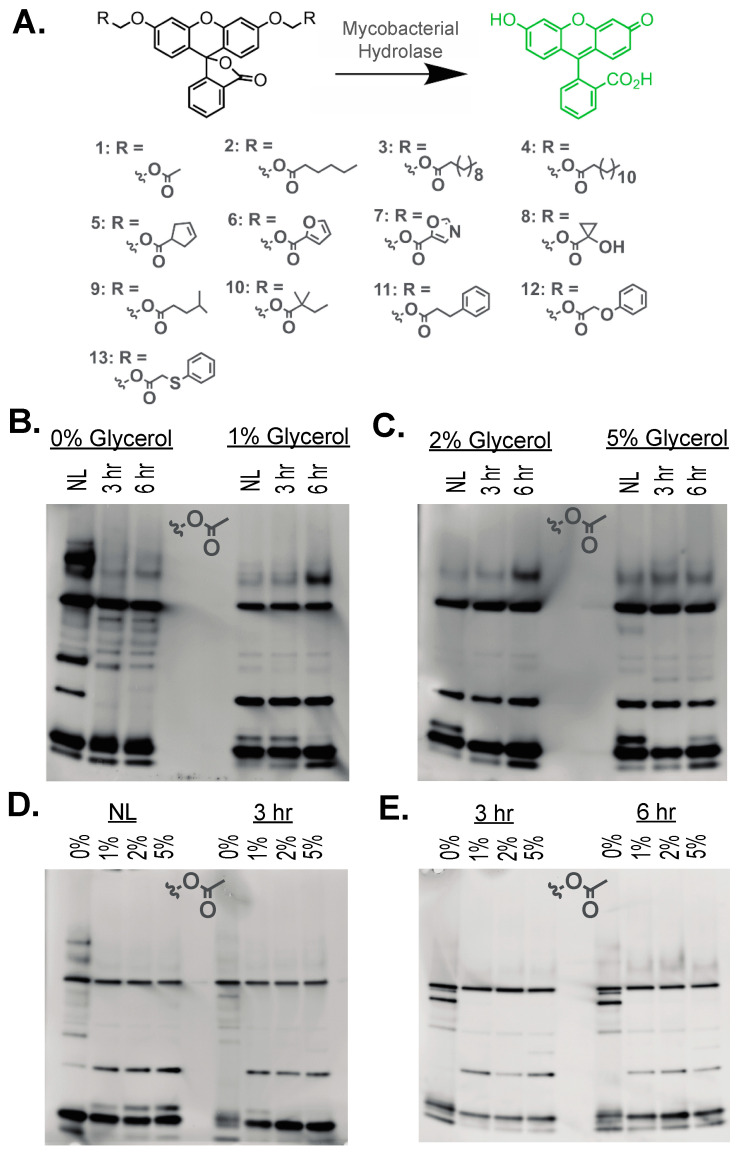
Fluorogenic substrates and in-gel hydrolase activity analysis. (**A**) Fluorogenic substrates. Hydrolysis of the ester-protecting groups (R) by a mycobacterial hydrolase releases the highly fluorescent product fluorescein. Ester functionalities of varying size and chemical properties were used to detect a wide array of serine hydrolases. Fluorogenic substrates are grouped based on common ester substrate classes. (**B**–**E**) In-gel serine hydrolase activity with substrate **1**. The in-gel activity assay showed serine hydrolases with differential activity across dormant and active growth states (NL = nitrogen limiting, 3 hr = 3 hour activation, 6 hr = 6 hour activation) in various glycerol concentrations (0%, 1%, 2%, and 5%). *Msmeg* lysates were separated using native-PAGE on a 4–20% polyacrylamide gel. Total protein (12 μg) was standardized by BCA assay. After native-PAGE (200 V for 105 min on ice), fluorogenic substrate **1** was added to the surface of the gel (10 μM; 5 μL of 10 mM substrate **1** in 5 mL PBS). After 15 min of exposure, bands were visualized using a LI-COR Odyssey^®^ M Gel imager. (**B**,**C**) Hydrolase activity changes based on dormant versus reactivated growth for each glycerol concentration. (**D**,**E**) Hydrolase activity changes with increasing glycerol concentration for each growth condition.

To compare hydrolase activity based on growth conditions (dormant versus active and versus carbon availability), *Msmeg* cultures were collected and gently lysed by sonication. *Msmeg* lysis used a previously optimized procedure for sonication that maximized recovered active serine hydrolase activity [19]. Total protein concentration was standardized (Figure S1) with consistent protein loading across samples (Figure S2). Active hydrolases in each lysate were then separated by native-PAGE, following an optimized procedure ensuring complete, reproducible separation (Figure 1A) [19]. To specifically visualize active hydrolases within each sample, hydrolase activity was detected by an in-gel enzymatic assay using a library of fluorogenic ester substrates (Figure 2A). In-gel enzymatic activity assays provide a rapid and sensitive methodology for visualizing and quantitating shifts in hydrolase activity with 50–100-fold higher sensitivity than ABPP and the ability to diversify hydrolase substrates [10,18,19,22].

For serine hydrolase substrates, we used a library of fluorogenic esters (Figure 2A) built upon acyloxymethyl ether fluorescein [19,23,24,25,26]. These substrates provide sensitive, rapid, and robust signals of hydrolase activity [19,27,28]. The substrates have low background hydrolysis due to the added hydrolytic stability of the AM ether bridge between the quiescent fluorescein and the reactive ester functionality [24]. By switching out the ester linkage, the substrate specificity of mycobacterial hydrolases can be visualized across growth conditions [19,25,26,27,28,29]. Substrates for investigation were chosen based on their ability to generally classify hydrolases based on substrate length (**1**–**4**), previous reactivity against mycobacterial hydrolases (**5**–**8**), and preliminary evidence of mycobacterial growth condition relevant reactivity (**9**–**13**) [19,25,26,27].

The in-gel fluorescent signal is only produced in the presence of a native-PAGE band corresponding to an active hydrolase that can catalyze the activation of that ester substrate (Figure 1A and Figure 2B–E). Previous disadvantages of in-gel enzyme measurements, including high signal-to-noise ratios and smeared bands, were minimized by the rapid and sensitive detection available with these fluorogenic substrates [18,19,30]. The relative enzymatic activities of individual hydrolases were visualized and quantitated based on the intensity of the bands where changes in enzyme activity could be compared across growth conditions and carbon availability (Figure 2B–E). Running multiple samples concurrently allowed for efficient observation of the changes in the activity of multiple serine hydrolases across *Msmeg* growth states.

Starting with the simplest acetyl ester substrate (**1**), significant numbers of active hydrolases were observed across dormant and reactivation conditions (Figure 2B,C), with the highest number of individual active hydrolases (>12 hydrolases) observed in the nitrogen limiting (NL) samples with no added carbon (0% glycerol). These hydrolases likely serve essential roles in lipid derivation and accumulation pathways necessary for the establishment and persistence of the dormant growth state. Compared to past in-gel measurements in *Msmeg*, our optimized methodology provides visualization of >2× more hydrolases against similar acetyl ester fluorogenic substrates [10,18,19,22]. The total number of observable hydrolases in our samples is also comparable with some ABPP substrates [11]. Unlike ABPP, our in-gel measurements also allow rapid comparison of hydrolase activity across 10 growth conditions per gel within an approximate 2 h period.

**Figure 3:**
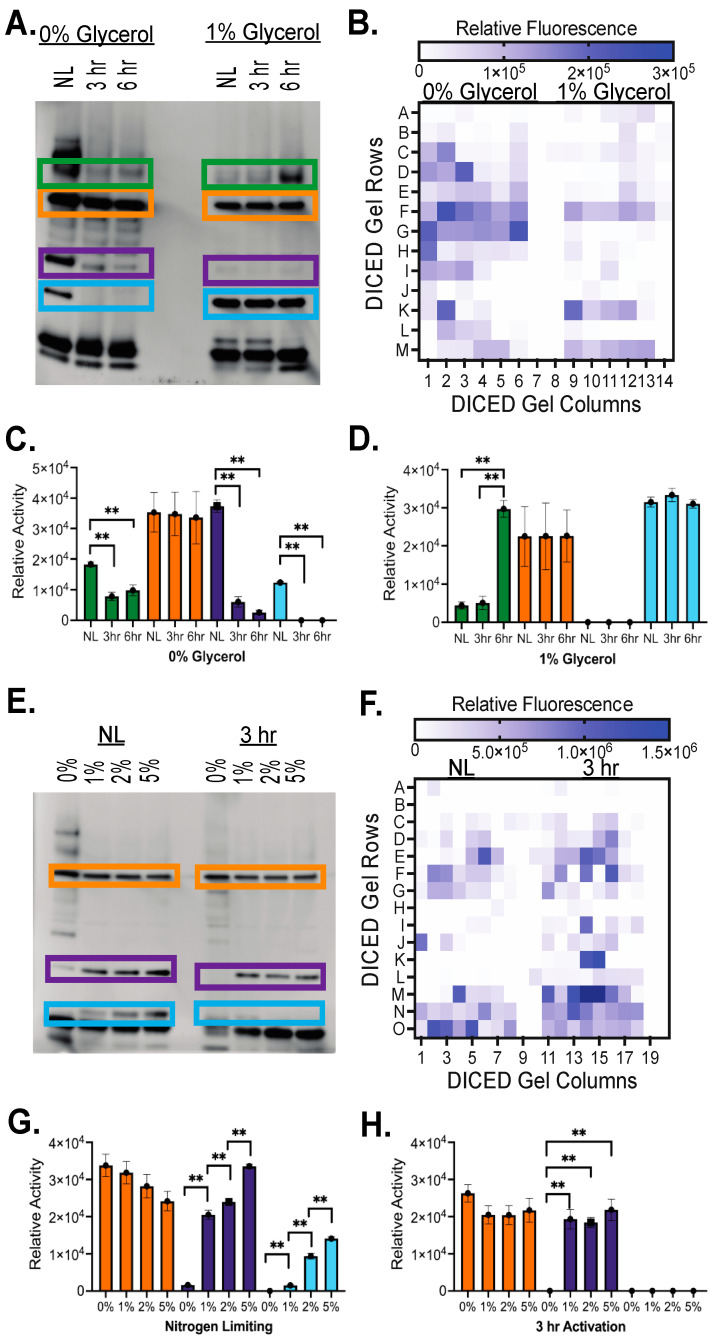
Quantitative comparison of hydrolase activity shifts. (**A**,**E**) Individual hydrolase bands were selected from in-gel enzyme measurements, and observed shifts in hydrolase activity were quantitated by ImageJ (**C**,**D**,**G**,**H**) or by DEG (**B**,**F**). In-gel measurements conducted as in Figure 2. (**C**,**D**,**G**,**H**) In-gel hydrolase activity quantified using ImageJ. Relative hydrolase activity within the boxed regions was calculated by measuring the pixel intensity of each region and subtracting the background pixel intensity within a control region on the same gel without hydrolase activity. The average intensity of three replicates is shown with SD. The relative activity is shown as each gel was imaged and its pixel intensity quantitated separately, allowing relative hydrolase activity comparisons within the same gel. A 2-tailed T-test assuming unequal variance was performed; the asterisks (**) represent significant differences (*p*-value < 0.01). (**B**,**F**) For DEG analysis, native-PAGE-separated samples were diced and sectioned into wells using a 384-well DEG cutter plate. Letters A–O represent the rows of wells the gel was diced into, while numbers 1–19 represent the columns. Substrate **1** (3 μM) in PBS was added, and the fluorescence (λ^Ex^ 488 nm; λ^Em^ 520 nm) measured every 1 min for 2 hr at 25 °C. The fluorescence of the background subtracted slope was calculated and plotted as a heat map (GraphPad Prism Ver10). The scale of the relative fluorescence change was adjusted to differentiate between high- and low-activity hydrolase gel fragments.

Visual comparisons across samples indicate observable shifts in hydrolase activity based on dormant versus active growth and based on carbon availability. Multiple high-intensity hydrolases were found constitutively active across growth conditions, while other hydrolase activity changes based on reactivation or carbon availability (Figure 2B–E). Picking multiple prominent bands for comparison based on reactivation (Figure 3A), relative hydrolase activity was quantitated based on band intensity and standardized across three replicates (Figure 3C,D). These highlighted bands within 0% and 1% glycerol illustrate common shifts observed in hydrolase activity. Like the orange band (Figure 3), many serine hydrolases were constitutively active and exhibited the same level of enzymatic activity in all growth conditions, indicating a general role for these serine hydrolases in standard metabolic functions. Compared to the 0% glycerol/NL conditions, multiple hydrolases (purple and blue bands) showed sequential or complete loss of activity upon reactivation, suggesting a necessity for increased hydrolase activity under nitrogen starvation or stress conditions that dissipate upon removal of these stress conditions. These hydrolases would likely not be required for the dormant to active growth transition needed for the persistence or activation of latent infections. Opposingly, increased serine hydrolase activity was also observed with increasing activation time, indicating hydrolases likely involved in lipid hydrolysis upon reactivation (green band). When comparing hydrolase activity based on carbon availability (Figure 3E–H), similar patterns emerge with constitutively active hydrolases (orange) and shifting hydrolase activity (purple and blue). For carbon availability, shifting hydrolases mostly exhibit increased activity with increased glycerol concentration (Figure 3G,H), providing more support in lipid accumulation. These shifts are largest in the nitrogen-limiting conditions, indicating high sensitivity to carbon usage under stress conditions (Figure 3G).

As further confirmation of activity shifts, native-PAGE gels were diced using a DEG cutter plate to provide rapid quantitation of activity changes [30,31,32]. For DEG assays, diced gel pieces were incubated with substrate **1**, the rate of ester hydrolysis was calculated, and the relative fluorescence rates were graphed. Due to the size of the wells in the 384-well plate, each row of wells is not necessarily representative of a single band on the gel [31,32]. Many hydrolase bands were diced between or across wells, leading to an inability to directly align in-gel and DEG analysis when comparing activity across single-diced wells, rows, or columns. Thus, we used in-gel analysis and DEG analysis as complementary techniques to analyze hydrolase activity, using DEG as confirmation of the patterns observed by in-gel analysis. For example, comparing the orange band across 0% and 1% glycerol with in-gel (Figure 3A) and DEG (Figure 3B) confirms the constitutive activity of this hydrolase with higher relative activity with carbon starvation (0% glycerol) than limited carbon availability (1% glycerol). DEG analysis also illustrates the higher total relative hydrolase activity in 0% glycerol (Figure 3A,B) and 3 h activation (Figure 3E,F) samples. Overall, combined in-gel and DEG analysis confirmed systematic shifts in activity for serine hydrolases active against the simplest acetyl ester substrate (**1**) based on dormant versus reactivation growth conditions and based on carbon availability.

### 2.3. Patterns of Hydrolase Activation across Ester Substrates and Serine Hydrolase Classes

**Figure 4:**
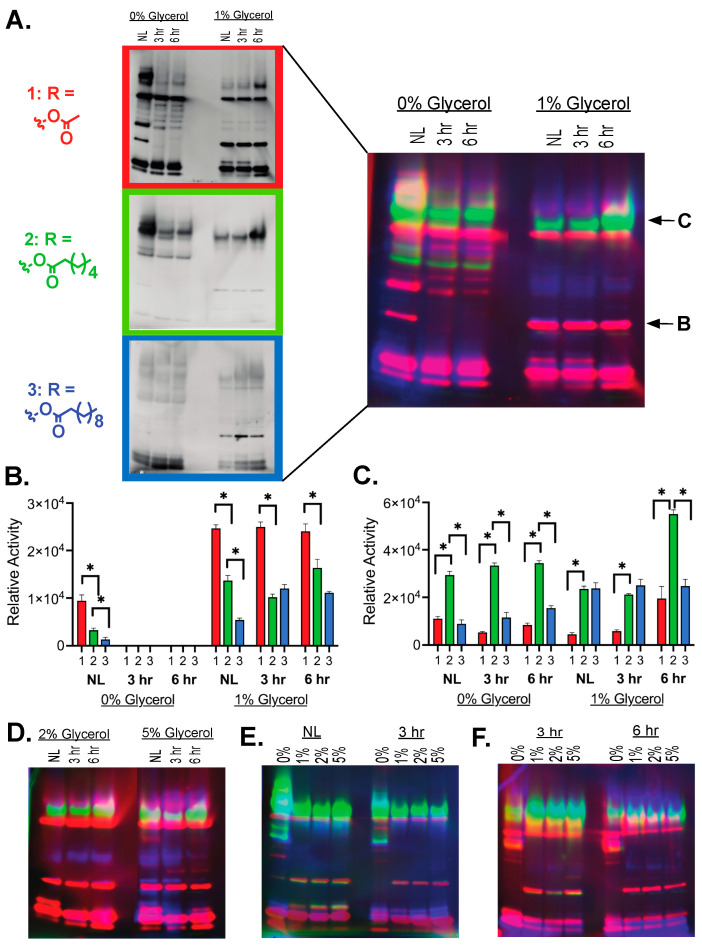
Substrate specificity comparison by overlayed in-gel analysis. (**A**) Comparative substrate specificity against substrates **1** (red), **2** (green), and **3** (blue). In-gel measurements conducted as in Figure 2. Images were overlayed in Adobe Photoshop Ver28. (**B**,**C**) In-gel hydrolase activity for labeled bands in (**a**). Quantitation performed identically to Figure 3. Asterisks (*) represent significant differences (*p*-value < 0.05). (**A**,**D**) Hydrolase activity changes based on dormant versus reactivated growth for each glycerol concentration. (**E**,**F**) Hydrolase activity changes with increased glycerol concentration.

Broad classifications of hydrolases can be made based on their selectivity for shorter (<10 carbon) or longer (>10 carbon) ester substrates with shorter preferences mainly associated with metabolic esterases and longer preferences associated with storage lipid lipases [9,33,34]. Previous in-gel measurements of *Mtb* showed the highest activity against shorter ester substrates, but there was the emergence of newly visible hydrolases with longer (>8 carbon substrates) ester substrates [18]. To add new layers regarding the substrate preferences of mycobacterial hydrolases, we measured the shifts in hydrolase activity versus substrate and versus growth conditions (nitrogen starvation and carbon availability), beginning with increasing carbon chain length (Figure 4A). Matching with previous measurements [18,19], the shortest acetyl ester substrate (**1**) had the most hydrolase bands present across growth conditions, but significant hydrolase activity was visible with the switch to a hexyl ester (**2**), decyl ester (**3**), and dodecyl esters (**4**; Figure S5). As longer ester substrates required longer exposure times, the broadening of lower-intensity bands was observed, but individual hydrolase bands were still discernable above the background (Figure 4A). The relative activation profile of various bands also shifted, with some bands showing the highest relative activity against longer carbon chain length ester substrates.

To visually represent these shifts in hydrolase activity based on ester substrate, fluorescence from each substrate was false colored based on substrate and the relative fluorescence intensity against multiple ester substrates overlayed (Figure 4A). This analysis indicated that individual serine hydrolases selectively cleave distinct fluorogenic substrates. Certain bands are only visualized when exposed to substrates of specific lengths or structures, evidenced by bands only appearing as a single color (Figure 4A). Other serine hydrolases had broader substrate specificity as they performed reactions with diverse substrates irrespective of size and structure, evidenced by overlapping colored bands (Figure 4A). Quantitating shifts in hydrolase activity versus substrate and growth conditions reinforce these changes where the hydrolase responsible for band B (Figure 4B) prefers shorter substrates but catalyzes measurable reactions with all three substrates. For band B, patterns of substrate activation remain consistent across growth conditions with the exception of 3 h and 6 h activation, where the relative differences between the C6 and C10 substrate activation are statistically insignificant (Figure 4B). As a counterpoint, the other quantitated band, band C, underscored a hydrolase with a preference for longer chain ester substrates with higher specificity for substrate **2** (Figure 4C). Similar to band B, this pattern of relative substrate activation is maintained across the majority of growth conditions with the exception of insignificant differences in longer carbon chains in 1% glycerol conditions. Comparing more widely based on growth conditions (Figure 4D–F), a higher proportion of hydrolases active against longer substrates (blue, substrate **3**) show greater shifts in hydrolase activity in nitrogen limiting versus reactivation growth (Figure 4D), reflecting their likely roles in the breakdown and construction of ILI stores. Globally, although a few new hydrolases show activity against only longer carbon substrates, the majority of hydrolases are active across all three alkyl ester substrates with varying degrees of substrate preference, making the simple acetyl ester substrate (**1**) a good model for measuring global changes in serine hydrolase activity.

**Figure 5:**
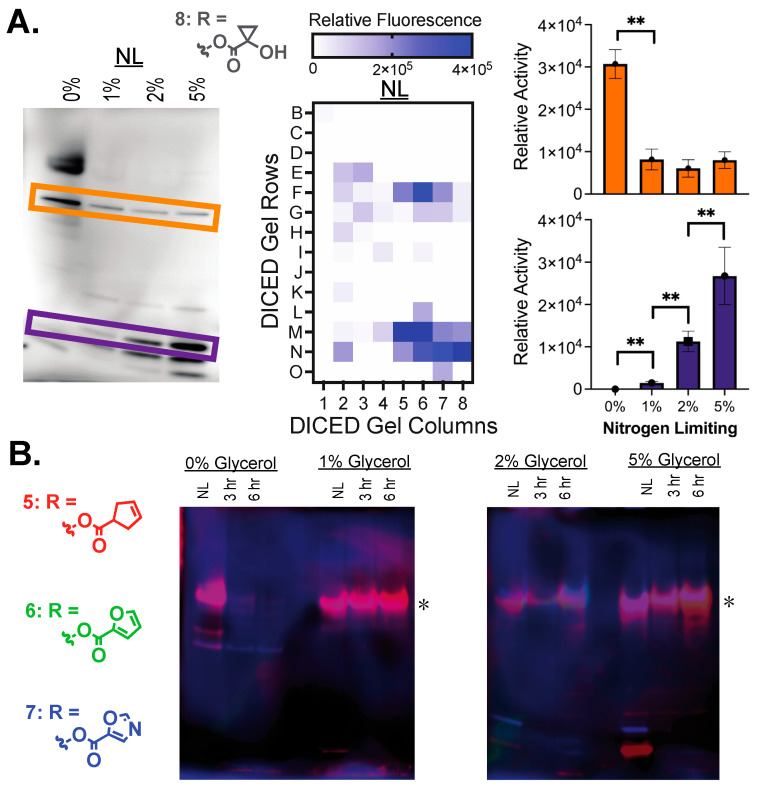
Expanded substrate comparisons by in-gel enzyme analysis. (**A**) In-gel analysis with the tertiary ester substrate **8**. In-gel analysis, DEG, and quantitation conducted as in Figure 3. Asterisks (**) represent significant differences (*p*-value < 0.01). (**B**) Molecular substrate specificity comparison by in-gel analysis against substituted cyclopentyl esters **5** (red), **6** (green), and **7** (blue). Substrate overlap conducted as in Figure 4. Hypothesized LipN band labeled with an asterisk (*).

Comparing more broadly across more chemically complex ester substrate classes (Figure 5 and Figures S3–S14), the broad activation pattern observed with the simple acetyl ester substrate (**1**) and other linear alkyl esters (**2** and **3**) is diminished with a smaller number of active esterases. For example, a tertiary cyclopropyl ester with inherent stability due to combined steric and stereoelectronic effects has limited activation with only three or four active hydrolases across all growth conditions (Figure 5A and Figure S9) [35,36]. These hydrolases, however, reinforce patterns of enzyme activity shifts observed with alkyl ester substrates, including (1) hydrolases only highly active under nitrogen limiting conditions and (2) hydrolases with opposing decreases and increases in activity based on carbon availability (Figure 5A). Additionally, small changes in substitution patterns across a cyclopentyl ester (**5**, **6**, **7**) illustrate the molecular specificity present within mycobacterial hydrolases, where an even smaller subset of active hydrolases is observed (Figure 5B). The addition of polar substituents into the ring further limits the enzyme activity, with only a single mostly constitutively active hydrolase being active (Figure 5, asterisk) against the oxazole ester (**7**) with the highest relative activity with high glycerol (2% and 5%). Previously, the mycobacterial hydrolase LipN was shown to have selective activity toward these cyclopentyl esters with high activity toward substrate **7** [26]. Using ABPP, LipN was also constitutively active across standard and stress growth conditions, which supports the assignment of this gel band as LipN (Figure 5, asterisk) [10,11]. With a lower number of active hydrolases, these more selective substrates provide molecular markers for individual hydrolases where shifts in the activity of this single hydrolase can now be followed across all growth conditions. Overall, in-gel enzyme measurements allow the rapid observation and quantitation of shifts in hydrolase activity and their ester substrate specificity across multiple growth conditions with tunable reactivity and enzyme selectivity based on ester substrate choice.

### 2.4. Identifying Individual Serine Hydrolases

**Figure 6:**
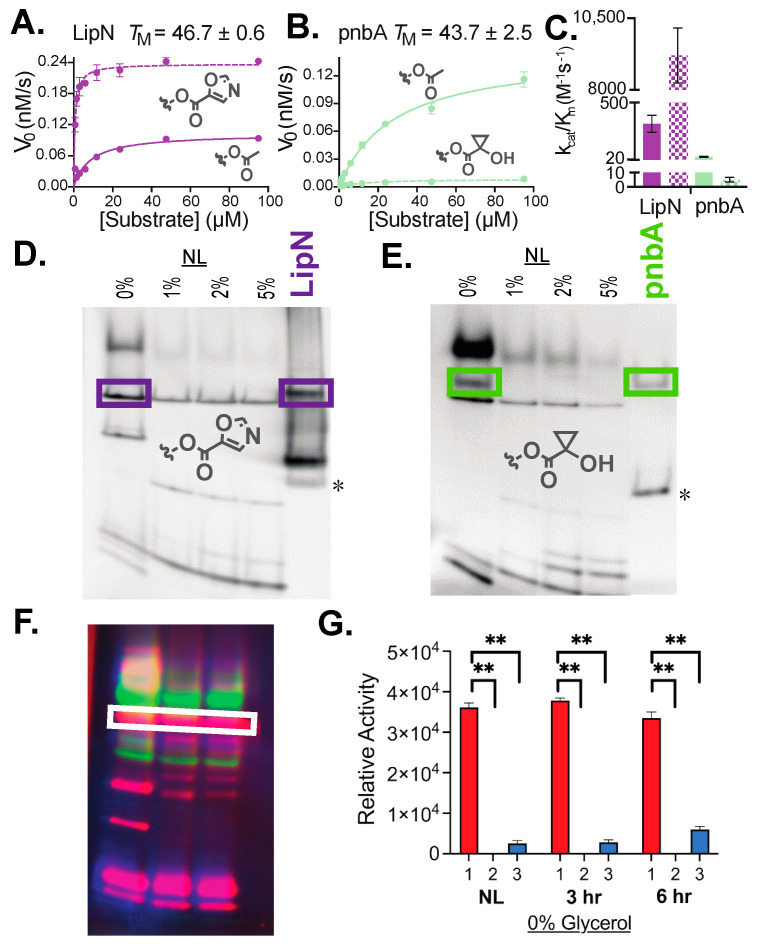
Preliminary assignment of in-gel enzyme activity using two purified mycobacterial hydrolases. (**A**–**C**) Confirmation of proper folding and catalytic activity for purified mycobacterial hydrolases. Initial reaction velocity against the labelled substrates was measured at a range of substrate concentrations and fitted to the Michaelis–Menten equation in GraphPad Prism. Overall catalytic efficiency (*k*_cat_/*K*_M_, (**C**)) was determined by extracting the individual kinetic constants. All values were determined in triplicate and are shown ± SD. (**D**,**E**) Measured in-gel activity of purified mycobacterial hydrolases matches with individual mycobacterial hydrolases present in various growth conditions. In-gel activity measured identically to Figure 2. Assigned band overlap shown by colored boxes. Asterisk (*) marks common, likely contaminating, *E. coli* band. (**F,G**) Quantitation of hypothesized LipN band based on activity against ester substrates **1** (red), **2** (green), and **3** (blue). Coloration and quantitation identical to Figure 4. Asterisks (**) represent significant differences (*p*-value < 0.01), with LipN strongly preferring shorter ester substrate **1**.

To begin to assign individual hydrolases to specific bands on the in-gel enzyme measurements, we chose two previously characterized mycobacterial hydrolases with unique substrate specificity patterns. Specifically, we focused on LipN, a mycobacterial hydrolase with high selectivity for an oxazole ester (**7**), which we hypothesized was responsible for the constitutive activity observed across growth conditions (Figure 5B) [26]. Additionally, we tested pnbA, the *Msmeg* homologue, to the promiscuous esterase BS2 from *Bacillus subtilis*. BS2 rapidly and selectively catalyzes the hydrolysis of tertiary cyclopropyl esters like substrate **8** [36,37]. The *B. subtilis* and *Msmeg* homologues of pnbA also have overlapping substrate specificity and functions [38,39]. After purifying the *Msmeg* homologues of LipN and pnbA, we confirmed their proper folding and measured their catalytic activity against the simple methyl ester (**1**) and their respective selective substrates (**7** and **8**, respectively; Figure 6A–C). Similar to previous measurements with a separate mycobacterial LipN homologue, *Msmeg* LipN has high enzymatic activity against the oxazole ester substrate (Figure 6A,C) [26]. *Msmeg* pnbA has significantly lower overall catalytic efficiency than LipN against substrate **1** but has measurable activity against the tertiary ester substrate **8** (Figure 6B,C).

After confirming the proper folding and catalytic activity of these two enzymes, purified enzyme samples were separated by native-PAGE alongside *Msmeg* lysates across carbon availability and visualized using their selective fluorogenic substrate (Figure 6D,E). For purified LipN, the highest intensity band matched with the predicted constitutively active *Msmeg* esterase band (purple boxes, Figure 6D), providing support to the assignment of this *Msmeg* esterase as LipN. A separate esterase is only active under nitrogen-limiting conditions with 0% carbon availability matched with purified *Msmeg* pnbA (green boxes, Figure 6E). Although both purified enzymes were >95% pure by reducing SDS-PAGE, an additional contaminating band (asterisk, Figure 6D,E) was common across both *E. coli* purified enzymes, suggesting with the higher sensitivity of the in-gel enzyme assay that an additional contaminating *E. coli* hydrolase was present in both purifications. An additional high-activity band was present in the LipN sample that may represent an additional *E. coli* contaminant or a separate isoform of *Msmeg* LipN, as mycobacterial LipN had monomer and dimer isoforms when purified from *E. coli* and had multiple post-translational modifications in *Mtb* [10,26]. With these additional contaminating bands, the two identified bands cannot be definitively assigned as LipN or pnbA. Yet, the combination of the overlap of these bands with previous activity measurements and with the unique substrate specificity of each serine hydrolase provides strong evidence for the positive assignment of these two esterase bands as LipN and pnbA. Further confirmation will be assisted by future CRISPR-based knockdowns combined with follow-up in-gel enzyme analysis [40].

## 3. Discussion

### 3.1. Serine Hydrolase Expression Shifts

The dynamic life cycle of mycobacteria with transitions between active, dormant, and reactivation growth complicates the diagnosis and treatment of tuberculosis [7,41]. Within mycobacteria, the essential role of ILIs for the survival and virulence of *Mtb* requires a battery of serine hydrolases and acyl-synthases underpinning the metabolic machinery catalyzing ILI formation and utilization [42]. These metabolic enzymes are required for the dormant versus reactivation switch and may represent a new antibacterial target for combatting persisters [4]. Targeting broad enzyme families like serine hydrolases is also an emerging strategy for overcoming the difficulty in developing new tuberculosis treatments and avoiding the rapid emergence of resistance [11].

Serine hydrolases lie at the intersection of lipid metabolism and mycobacterial growth transitions [8]. Mycobacteria contain a diversity of active serine hydrolases, with over 70 total hydrolases identified across multiple ABPP studies [10,11,13,14,15,16]. At least 11 of these serine hydrolases were labeled as priority therapeutic targets based on confirmed shifts in growth morphology upon treatment with 1,2,3-triazole urea inhibitors [11]. Differential hydrolase expression was also observed by ABPP across various growth conditions (acidic pH and hypoxia) [10,11,13,14]. Yet, given the time and sample scale required for ABPP, these studies only provided a snapshot of hydrolase activity within a single time and growth condition. ABPP studies have also varied the targeting ligand, but all current ligands shared similar chemical characteristics, being highly hydrophobic with extended alkyl chains linking the covalent targeting moiety to the enrichment motif [10,11,13,14,15,16].

To rapidly measure the dynamic, substrate-dependent shifts in serine hydrolase activity, we combined a robust mycobacterial growth system of nitrogen limitation, variable reactivation times, and carbon availability with nimble in-gel fluorogenic enzyme measurements. This growth system uses the nontoxic, fast-growing mycobacterial *Msmeg*, which undergoes the same dormant to reactivation transitions as *Mtb*, contains most metabolic hydrolases (9 out of 11 high-priority hydrolase targets), has a conserved pathway for TAG synthesis and accumulation, and simplifies the analysis with its increased growth rate [5,11,19,43]. Matching with previous measurements, nitrogen limitation led to a large increase in TAG formation (3.5–4X), followed by time and carbon availability-dependent TAG breakdown upon reactivation (Figure 1) [5]. Reactivation was previously seen to be rapid and linear with near complete TAG depletion 24 h post reactivation [5]. Thus, we chose to focus on the initial quartile of this reactivation period (<6 h), taking two time points (3 h and 6 h) for comparison, as we wanted to capture the dynamic wave of hydrolases produced upon initial reactivation. Across these two time points, statistically significant decreases in TAG levels were measured for intermediate carbon availability (1% and 2% glycerol) with rapid initial degradation followed by stabilization of TAG levels after 6 h.

Only two intracellular mycobacterial lipases have been directly connected to ILI breakdown, and only one of those hydrolases is present in *Msmeg* (MSMEG_3767) [5,44]. Yet, a significant number of hydrolases are active during reactivation (Figure 2B,C), especially in the 1% and 2% glycerol conditions where TAG levels significantly decreased (Figure 1C). These hydrolases undergo carbon and reactivation-dependent shifts in catalytic activity (Figure 3), suggesting regulated expression and/or post-translational control of their enzymatic activity. Complex regulation of mycobacterial hydrolase activity was previously proposed based on post-translational modifications (acetylation and phosphorylation) of essential catalytic amino acids in multiple mycobacterial hydrolases [10]. The proposed central TAG hydrolase in *Mtb*, LipY, is not present in *Msmeg*, but the orthogonal introduction of LipY expression did not significantly change TAG hydrolysis, further supporting our in-gel results showing that a range of serine hydrolases are involved in this process in *Msmeg* and likely throughout mycobacteria [5]. This diversity of hydrolases responsible for ILI and TAG hydrolysis is further reinforced by the differential hydrolase activity observed across variable ester substrates (Figure 4, Figure 5, and Figures S3–S13).

Increasing the alkyl chain length within the fluorogenic ester substrates allowed the visualization of distinct hydrolase activity likely corresponding with lipases most responsible for lipid breakdown (Figure 4) [18]. Even given the dependence of mycobacteria on ILI and TAG for energy and survival, the highest hydrolase activity was still measured for shorter chain ester substrates (2-carbon, red, **1**) with limited hydrolases preferring medium-chain substrates (6-carbon, green, **2**) and only minimal hydrolases with a strong preference for the longest chain substrate (10-carbon, blue, **3**) even with extended exposures for the longer substrates (Figure 4A). Although lipid profiles were complex, TB patients had higher proportions of shorter chain TAGs and free fatty acids (FFAs) in their blood than control patients, potentially reflecting this mycobacterial preference for shorter chain lipids [45]. Further shifting to more chemically complex ester substrates (Figure 2A) provided markers for individual serine hydrolases, supporting the diversity of metabolic hydrolase activity present in mycobacteria (Figure 5).

Unlike previous serine hydrolase activity measurements, which only showed decreased catalytic activity across variable growth conditions, our nitrogen limitation, reactivation, and carbon availability conditions all showed hydrolases with increasing, decreasing, and constitutive catalytic activity (Figure 2, Figure 3, Figure 4 and Figure 5) [10,19]. Nitrogen limitation was the strongest environmental initiator for ILI formation, leading to the most robust shifts in serine hydrolase activity [5]. Even though previous hypoxia and nutrient starvation measurements led to ILI breakdown, serine hydrolase activity was only downregulated [10,19]. Importantly, our nitrogen starvation system displayed the expected full, dynamic range of upregulated, downregulated, and constitutively active hydrolases independent of the ester substrate (Figure 4 and Figure 5). An additional novel feature of our data set is the robust expression of hydrolase activity under the highest stress level (nitrogen limitation and 0% glycerol; Figure 2 and Figure 3), with the highest number of hydrolases observable under these conditions. This expanded hydrolase activity likely reflects the induction of multiple metabolic pathways scavenging for energy to survive under this high stress. The unique hydrolases present under these high stress conditions (nitrogen limitation and 0% glycerol) could represent novel drug targets for combination treatment with current front-line therapeutics when mycobacteria are already experiencing high stress [46].

### 3.2. Preliminary Assignment of Individual Hydrolase Bands

Starting to deconvolute the complex serine hydrolase activity present under nitrogen limitation, reactivation, and carbon availability, we expressed and purified two *Msmeg* hydrolases and compared their hydrolase activity with native mycobacterial hydrolases by in-gel analysis (Figure 6). These two hydrolases, LipN and pnbA, were chosen based on their selective and divergent substrate specificity from previous broad ester substrate specificity screens, with LipN preferring oxazole esters (**7**) and pnbA catalyzing reactions with highly stable tertiary esters (**8**, Figure 2A) [26,38,39]. Purified *Msmeg* versions displayed the expected substrate preferences (Figure 6C) and in-gel activity against these substrates (Figure 6D,E).

Satisfyingly, the major purified LipN band overlapped with a constitutively active serine hydrolase present across growth conditions and shorter ester substrates (Figure 6D). From previous ABPP measurements, LipN was found to be active across variable growth conditions (low pH, hypoxia, and normoxia) and to be active across multiple ABPP ligands [10,11]. Additionally, a broad ester screen with LipN showcased a highly active metabolic esterase with preferences for short (<6 atoms) substrates and for cyclopentyl ester substrates [26]. Comparing this assigned LipN band across variable alkyl substrates further supports this assignment, as this band had high activity against the 2-carbon substrate (**1**) and significantly lower activity against the 6-carbon (**2**) and 10-carbon substrates (**3**) (Figure 6F,G).

Similarly, the major band for purified pnbA activation against substrate **8** matches with a single mycobacterial hydrolase band that is differentially expressed under the highest stress conditions (nitrogen limitation and 0% glycerol) (Figure 6E). Although its homologue in *B. subtilis* has been confirmed to have high activity in vivo, mycobacterial pnbA was not labeled in previous global ABPP measurements, potentially reflecting the larger, hydrophobic nature of ABPP ligands and the narrow substrate preferences of pnbA [11,38,39]. Additionally, the expression of pnbA under only a narrow set of growth conditions, outside the conditions tested in previous ABPP measurements, would have also limited its labeling. The differential expression and substrate preference of pnbA may be useful for prodrug targeting in mycobacteria, as *Msmeg* pnbA substrate preference is orthogonal to other bacterial hydrolases [38,39].

Although these two assigned mycobacterial lysate bands match the expected substrate preference of these two hydrolases, further experimentation will be required to definitively assign these two hydrolases. Complicating these assignments is the presence of additional bands within the purified hydrolase lanes and the potential for post-translational modification of endogenous versions of these hydrolases, which would shift the relative native-PAGE retention in comparison to the *E. coli* purified forms. Currently, we are working to validate CRISPR constructs across expected mycobacterial hydrolases to look for the downregulation or disappearance of individual bands upon CRISPR treatment, but the breadth of potential mycobacterial hydrolases has made this into a separate but connected investigation [40]. The eventual assignment of each band on these in-gel analyses will allow systematic tracing of shifts in the catalytic activity of individual hydrolases across growth conditions. The substrate specificity of all mycobacterial hydrolases could also be measured simultaneously, allowing rapid identification of orthogonal ester moieties applicable for prodrug development [39,47].

### 3.3. Advantages and Limitations of In-Gel Hydrolase Analysis

Serine hydrolases are a broad class of conserved enzymes with essential functions in metabolism, immune regulation, neurosignaling, detoxification, and signal transduction [9,33,48]. With over 200 enzymes in humans, studying the overlapping and complex regulation of hydrolase activity requires precise molecular tools [33]. Fluorogenic ester substrates have been a valuable tool for dissecting this complex serine hydrolase activity across organisms, applications, and hydrolase classes and for developing diagnostics for pathogen and cancer detection with tunability based on activating group and fluorophore [41,49,50,51]. For our fluorogenic esters, we used a library of fluorescein derivatives with acyloxymethyl ether activating groups [24]. Fluorescein-based esters are validated probes for the detection of metabolically active *Mtb* in sputum, the analysis of bacteria in wastewater, and the differentiation of live versus dead cells [49]. Our library of fluorogenic esters provides sensitive detection of hydrolase activity with low background hydrolysis and high selectivity based on ester functionality [52]. Across multiple purified mycobacterial hydrolases, switching out the activating ester functionality provided selective probes for mycobacterial hydrolases and tools for understanding their key substrate specificity determinants [25,26,27,28,29].

Combining these fluorogenic esters with native-PAGE separation provides a rapid, customizable, and sensitive detection method for serine hydrolase activity. Using fluorogenic in-gel analysis, mycobacteria hydrolases were previously shown to be dynamically active based on growth conditions (hypoxia and nutrient limitations) and macrophage infection with similar hydrolase activity observed across *Mtb* and *Msmeg* [10,18,19,22]. Unlike past in-gel analysis, which showed only 3–4 active hydrolases irrespective of growth conditions or ester substrate, the combination of nitrogen limitation with shifting carbon availability provided an expanded repertoire of active hydrolases with 12+ hydrolases active across variable growth conditions and ester substrates [19]. Additionally, previous in-gel hydrolase analysis based on hypoxia and nutrient limitation only showed decreased hydrolase activity, but nitrogen limitation and shifting carbon availability provided the full range of serine hydrolase activity shifts with multiple hydrolases have increasing, decreasing, and constitutive activity dependent on carbon availability and dormancy versus active growth [10,19].

A major advantage of in-gel hydrolase analysis over previous ABPP measurements is the ability to rapidly analyze a range of substrates [10,19]. Since previous ABPP studies have all utilized long lipid-like substrates, a previous switch to a shorter two-carbon ester substrate like **1** produced an additional active hydrolase not observed by any ABPP study [18]. ABPP studies observed a higher number of active hydrolases than our in-gel measurements, but each of the studies only used a single hydrophobic substrate and a maximum of four experimental growth conditions [10,11,13,14,15,16]. The ability to rapidly measure dynamic changes in hydrolase activity across a range of growth conditions, against a wider range of hydrolase substrates, and with 50–100 fewer samples makes in-gel analysis a valuable complementary technique to current ABPP screens [10]. Other advantages of in-gel enzyme analysis are the ability to identify multiple hydrolase targets concurrently, to study functional enzyme shifts, and even to provide global measurements of the substrate specificity of individual hydrolases [19,53]. More chemically complex substrates like **7** and **8** also provide selective detection reagents for individual hydrolases and could be used to develop ester-hydrolase pairs for mycobacterial selective antibiotic development [39,54]. Similar sulfatase active fluorogenic substrates combined with in-gel analysis provided molecular fingerprints based on mycobacterial species and even *Mtb* strains and confirmed the high sensitivity of fluorogenic probes for mycobacterial detection [20].

In our in-gel analysis, we added new layers of comparison versus past versions by presenting overlapping substrate specificity differences and by combining comprehensive in-gel analysis with confirmatory diced gel electrophoresis (DEG) [30,31]. DEG and in-gel analysis provide high sample reproducibility and high sensitivity, with the two methodologies amenable to rapid screening for chemical inhibitors and novel enzyme identification [55,56,57]. Showing the power of these combined techniques, a recent study on breast cancer cell lines using fluorogenic glycosidase substrates across multiple cancer cell lines identified α-mannosidase C21 as a selective marker for breast cancer [55]. Similar multiplexing of our in-gel analysis and DEG across mycobacterial growth conditions will be used in the future to identify dormant or reactivation selective hydrolases that can be used as diagnostic or inhibitor targets. In our current analysis, DEG was used as a confirmatory follow-up to in-gel measurements where major hydrolase activity bands and shifts in hydrolase activity across growth conditions overlapped between in-gel and DEG measurements. In future studies, a switch to a gel size with dimensions that match the DEG plate (4.5 mm square pieces) would increase the direct overlap between in-gel and DEG signals [31,32].

Although a powerful complementary technique to ABPP and DEG, our in-gel analysis has limitations. Detection of individual hydrolases is limited by the diffusion of activated fluorogenic substrates within the polyacrylamide matrix [30,51,53]. For high-activity substrates like **1**, where activation is rapid and lower exposure times are required, limited diffusion is observed, and bands remain sharp and distinct. Yet, with longer and less active substrates like **3** and **7**, broader and more diffuse bands are observed due to the extended time required for visualization. Even with this diffusion, limited bleeding between individual bands is observed, allowing for the assignment of individual hydrolase bands across growth conditions and samples. Unlike ABPP, whose methodology standardly contains integrated mass-spectrometry identification, our in-gel analysis provides global patterns of hydrolase activity shifts across multiple substrates and growth conditions but requires follow-up experiments to assign individual bands to their underlying hydrolases. Past in-gel analysis used gel excision followed by trypsin digestion and mass spectrometry to propose potential hydrolases responsible for each in-gel band [10,19]. Utilizing this workflow on various in-gel bands from our current analysis yielded a complex mixture of mycobacterial proteins and potential hydrolases (Figure S16). We think the difficulty in applying this methodology in our current study is due to the high sensitivity of the fluorogenic substrates, which allows the visualization of minor components and the low sample quantity loaded onto each lane. Future analysis would benefit from the two-dimensional native-PAGE separation used to identify enzymes like the cancer-selective glycosidase, α-mannosidase C21 [55]. As a complementary technique to mass spectrometry confirmation, we used purified versions of two mycobacterial hydrolases with known selective substrate specificity to preliminarily identify the bands corresponding to these two mycobacterial hydrolases (Figure 6).

## 4. Materials and Methods

### 4.1. Cell Culturing

*Msmeg* was grown in Middlebrook 7H9 medium, Sigma Aldrich, St. Louis, MO, USA (10 mL) containing 0.05% tween-80 and 0.2% glycerol at 37 °C and 225 rpm. The medium was inoculated to an initial OD_600nm_ of 0.05 and incubated at the above conditions until the OD_600nm_ reached 1–1.5. The cells were harvested through centrifugation (5000× *g*, 10 min) and washed twice with sterile Phosphate Buffer Saline (PBS; 80 g NaCl, 2 g KCl, 14.4 g Na_2_HPO_4_, and 2.4 g KH_2_HPO_4_ in 1 L H_2_O; pH = 7.4) containing 0.05% Tween-20 and resuspended in PBS at OD_600nm_ = 10. The washed *Msmeg* culture was used to inoculate new cultures (1 L) at an initial OD_600nm_ of 0.05–0.1 with varying concentrations of glycerol (0%, 1%, 2%, and 5%, *v*/*v*) in a nitrogen-limiting mineral salt medium (MSM NL) (2 g/L Na_2_HPO_4_, 1 g/L KH_2_PO_4_, 0.5 g/L NaCl, 0.2 g/L MgSO_4_, 20 mg/L CaCl_2_, and 0.05 g/L NH_4_Cl) to mimic the dormant state that Mtb generally adopts upon original infection [5]. Tyloxapol was added at a final concentration of 0.02% (*v*/*v*) to prevent bacterial cell clumping, and cultures were allowed to grow for 48 h. These cultures were each split into three separate subcultures, and growth conditions were shifted to mimic various time points post-reactivation. All subcultures were centrifuged, and 2 of the 3 pellets from each glycerol concentration were resuspended in reactivation media (250 mL). One culture was allowed to reactivate for 3 h, another reactivated for 6 h, and the final was not subjected to new conditions. Reactivation was accomplished by growing *Msmeg* in MSM with sufficient nitrogen (1 g/L NH_4_Cl) after removal from MSM NL [5].

### 4.2. Cell Pellet Collection and Lipid Extraction

Cell samples were centrifuged (4000× *g*, 10 min, 4 °C) and washed with sterile water (3×). Pellets were lyophilized overnight and weighed to determine the exact dry weight of the mycobacterial residue. Lipids were then extracted using 2 mL of MeOH-0.3% NaCl (10:1 *v*/*v*) per 50 mg dry extract [5]. Petroleum ether (1 mL) was added, and the solution was rocked at room temperature for 15 min. The mixture was centrifuged (4000× *g*, 5 min), and the upper organic layer containing extracted lipids was transferred to a fresh pre-weighed vial. The petroleum ether extraction was repeated 3 times with a final centrifugation step of 4000× *g* for 15 min. The solvent was removed via lyophilization, and the dry lipid weight was obtained. Lipids were resuspended in dichloromethane (200 μL).

### 4.3. Analysis by Thin Layer Chromatography

Extracted lipids were compared by thin-layer chromatography to follow shifts in the relative concentrations of TAG in each sample. Petroleum ether/diethyl ether (90:10 *v*/*v*) was used as the solvent/mobile phase. Lipid samples were spotted on a silica TLC plate alongside a TAG control (Triolein 18:1; 1,2,3-tri-(9Z-ocatdecenoyl)-glycerol; Avanti Polar Lipids, Alabaster, AL, USA); the amount added was standardized according to the dry weight of mycobacterial residue [5]. After TLC, lipids were visualized on the plate by submerging in a solution of 10% phosphomolybdic acid in absolute ethanol and heating on a hot plate. Using ImageJ software, the relative amounts of TAG in each sample were quantized, and statistical analyses were performed.

### 4.4. Msmeg Lysis and Measuring Protein Concentration

*Msmeg* cells were lysed to release protein contents in solution using sonication. Samples were diluted in PBS (500 μL) and subjected to 4 rounds of sonication (60 Hz, 30 s) with sample inversion and rest on ice between each round (60 s) [19]. Once proteins were released from the cells, the protein concentration in each sample was measured using a bicinchoninic acid (BCA) protein assay using bovine serum albumin (BSA) for standardization. The concentrations of sonicated *Msmeg* protein samples were measured in duplicates in a 96-well microplate. After cooling to room temperature, the absorbance at 562 nm was measured using a plate reader and the concentrations were calculated according to the standard curve.

### 4.5. Native-PAGE

Lysates were separated using native-PAGE (Thermo Fisher Scientific, Waltham, MA, USA). Protein concentrations were standardized (12 μg protein; total volume = 20 μL in PBS; 5 μL 4× native-PAGE sample buffer (62.5 mM Tris HCl, 25% glycerol, 1% Bromophenol Blue; pH = 6.8)) for the various growth states. Each of the prepared samples (20 μL) was added to the wells on a Tris-Glycine polyacrylamide gel (4–20%). Gels were run in 1× native-PAGE running buffer (25 mM Tris, 192 mM Glycine) at 200 V for 105 min on ice [19].

### 4.6. Fluorogenic Ester Substrate Exposure and Gel Imaging

Active serine hydrolases in each native-PAGE gel were visualized using fluorogenic ester substrates in PBS and imaged using a LI-COR Odyssey M Imager (Lincoln, NE, USA). Ester substrates were added at various concentrations, depending on the relative activation of those substrates: substrates **1**–**4** (3 μM), substrates **9**–**10** (6 μM), and substrates **5**–**8**, **11**–**13** (12 μM) (Figure 2A). Substrate solution was added directly to the gel surface and scanned immediately with additional scans performed at 10 min increments for up to 40 min using the 488-channel program on the imager (excitation: 488 nm, emission: 519–543 nm). Exposure times and amount of added substrate varied due to differing levels of activation and the time required for observation.

### 4.7. Diced Gel Assay (DEG)

After native-PAGE analysis, the gel was sandwiched between the two halves of a Saniome DEG Cutter-plate (Cosmo Bio USA, Carlsbad, CA, USA) and centrifuged (3000 rpm, 5 min, 23 °C) to cut the gel and separate individual pieces into 384 wells [31,32]. A prepared fluorophore solution (80 μL total volume in PBS) for the various types of fluorogenic substrates, substrates **1**–**4** (3 μM), substrates **9**–**10** (6 μM), and substrates **5**–**8**, **11**–**13** (12 μM), was added to each well. Fluorescence (excitation = 485 nm, emission = 520 nm) was measured using a Synergy H1 Hybrid Reader (Biotek, Agilent, Winooski, VT, USA) over the course of 2 h at 1 min intervals [52]. Slopes were fitted to a linear equation, averaged across duplicate runs, and background corrected based on wells containing only fluorogenic substrate and PBS.

### 4.8. LipN and pnbA Expression and Purification

Two mycobacterial proteins with conserved serine hydrolase activity, LipN and pnbA, were expressed in competent *E. coli* cells (C41). Plasmids encoding LipN (pET-28a(+): LipN *M. smegmatis*; GenBank WP_014877378) and pnbA (pET-28a(+): pnbA *M. smegmatis*; GenBank WP_011729246.1) were synthesized by Genscript and inserted between NdeI and XhoI sites in pET-28a(+) with the N-terminal histag in frame. Plasmids were freshly transformed into *E. coli* C41 cells and grown in overnight cultures in LB containing 40 μg/mL kanamycin at 37 °C and 225 rpm. Saturated overnight cultures were used to inoculate 1 L LB cultures (1:100 dilution). Expression cultures were grown at 37 °C and 225 rpm for 4–6 h until the cultures reached full density. Expression was induced with the addition of Isopropyl β-D-1-thiogalactopyranoside (IPTG) (1 mM). Each culture continued incubation with constant shaking (225 rpm) for 16–18 h with pnbA induction at 20 °C and LipN induction at 37 °C [26,36,37]. Bacterial cultures were collected by centrifugation (6000× *g* for 10 min at 10 °C), pellets resuspended in PBS, and pellets frozen at −20 °C prior to purification.

Nickel-metal affinity chromatography was used to purify each protein. Cell pellets were lysed using an EmulsiFlex-C3 homogenizer (Avestin, Ottawa, ON, Canada) cycling at 20,000–25,000 psi for 2 min and the lysate was centrifuged (25,000× *g*, 10 min, 10 °C). Ni-NTA (nickel-nitrilotriacetic acid; GoldBio; 1 mL) was added to the supernatant, and the solution was mixed for 30 min on a rotor at 4 °C. The Ni-NTA-protein solution was added to a drip column, and the flow through was discarded. The column was washed twice (30 mL each) with PBS containing 10 mM imidazole and once (30 mL) with PBS containing 25 mM imidazole. Purified protein was eluted by adding PBS containing 500 mM imidazole (1 mL) to the capped column and incubating for 5 min before collection. The eluted proteins were then dialyzed against PBS at 4 °C for 1 day, and their final concentrations were measured by UV absorbance (LipN extinction coefficient = 36,900 M^−1^ cm^−1^; pnbA extinction coefficient = 84,005 M^−1^ cm^−1^) using the Take 3 plate on Synergy H1 multimode plate reader (Biotek, Agilent, Winooski, VT, USA). Purified protein purity was confirmed by SDS-PAGE on 4–20% acrylamide gels stained with colloidal Coomassie brilliant blue; theoretical MW = 40.7 kDa LipN; MW = 56.7 kDa pnbA.

### 4.9. Thermal Stability

The melting temperature (*T*_m_) for each protein was determined by measuring the change in Sypro Orange fluorescence during thermal denaturation in a real-time PCR instrument [58]. A solution of protein (0.6 mg/mL), PBS, and Sypro Orange dye (Thermo Fisher Scientific, Waltham, MA, USA) (1:500 dilution) was added to 3 wells on a low-profile PCR plate and mixed. The plate was loaded into a Bio-rad C1000 Thermocylcer (Bio-Rad, Hercules, CA, USA) with the CFX96 Real-time System and was heated from 15 °C to 95 °C at 1 °C/min. Fluorescence was measured every second to obtain a melting curve for each protein, and the normalized first derivative of each curve was analyzed.

### 4.10. Enzymatic Activity Analysis

The enzymatic activities of each protein were measured against various fluorogenic ester substrates [26,52]. Each fluorogenic ester substrate was 1:1 serially diluted eight-fold with PBS containing acetylated BSA (pH 7.4 phosphate-buffered saline, 0.1 mg/mL acetylated bovine serum albumin; 95 μL) and incubated with either LipN (5 μL, 1.5 μg/mL, 36.9 nM in PBS + BSA) or pnbA (5 μL, 5 μg/mL, 123 nM in PBS + BSA). Fluorescence (excitation = 485 nm, emission = 520 nm) was then measured every 15 s on a Biotek Synergy multimode plate reader over the course of 4 min at 25 °C. A standard curve of fluorescence vs. fluorescein concentration was obtained by measuring the fluorescence of 1:1 fluorescein (0–300 nM) standard dilutions constructed in PBS+BSA. The resulting standard linear equation was used to calculate the initial reaction velocities. Initial velocities were then fitted to the Michaelis–Menten equation using GraphPad Prism, and kinetic parameters were calculated.

### 4.11. Native-PAGE of Purified Proteins

Purified proteins (LipN = 1 μg, pnbA = 6 μg) were analyzed alongside *Msmeg* lysate samples by native-PAGE. Fluorogenic substrates that LipN (substrate 7) and pnbA (substrate 8) were found to be active via the kinetics analysis and were used for best sensitivity. The distance traveled by endogenous serine hydrolases was compared with that of purified LipN and pnbA proteins to identify potential matches.

## Data Availability

Dataset available on request from the authors.

## Supplementary Materials

The following supporting information can be downloaded at https://www.mdpi.com/article/10.3390/molecules29143386/s1, Figures S1–S16; Protein concentration standardization, Expanded in-gel measurements with additional ester substrates, Thermal stability, Kinetics, and Preliminary MS assignment.
