# Supplementary material for: Shifting Mycobacterial Serine Hydrolase Activity Visualized Using Multi-Layer In-Gel Activity Assays
Source: Molecules. 2024 Jul 18;29(14):3386. doi: 10.3390/molecules29143386 (PMC11279797; doi:10.3390/molecules29143386)
Supplement: Supplementary file 1 [file molecules-29-03386-s001.zip › molecules-3091857-supplementary.pdf]

Shifted mycobacterial esterase activity visualized under dormant versus active growth conditions using multi-layer in-gel activity assays

Allison L . Goss[1], Renee E. Shudick[1], R. Jeremy Johnson [1,*]

[1] Department of Chemistry and Biochemistry, Butler University, Indianapolis, IN 46208

[*] Correspondence: rjjohns1@butler.edu; Tel.: 1-317-940-9062

Supplemental Information

Supplemental Tables and Figures

Figure S1:	BCA Assay Standard Curve.	S2
Figure S2:	Total Protein Loading Native-PAGE Samples.	S3
Figure S3:	In-gel Enzyme Analysis with Substrate 2.	S4
Figure S4:	In-gel Enzyme Analysis with Substrate 3.	S5
Figure S5:	In-gel Enzyme Analysis with Substrate 4.	S6
Figure S6:	In-gel Enzyme Analysis with Substrate 5.	S7
Figure S7:	In-gel Enzyme Analysis with Substrate 6.	S8
Figure S8:	In-gel Enzyme Analysis with Substrate 7.	S9
Figure S9:	In-gel Enzyme Analysis with Substrate 8.	S10
Figure S10:	In-gel Enzyme Analysis with Substrates 9 and 10.	S11
Figure S11:	In-gel Enzyme Analysis with Substrate 12.	S12
Figure S12:	In-gel Enzyme Analysis with Substrates 11 and 13.	S13
Figure S13:	Overlayed In-Gel Analysis of Substrates 5, 6, 7.	S14
Figure S14:	LipN and pnbA Melting Curve.	S15
Figure S15:	Michaelis Menten Parameters for LipN and pnbA	S16
Figure S16:	Potential Serine Hydrolase Identities Determined by Mass Spectrometry	S17

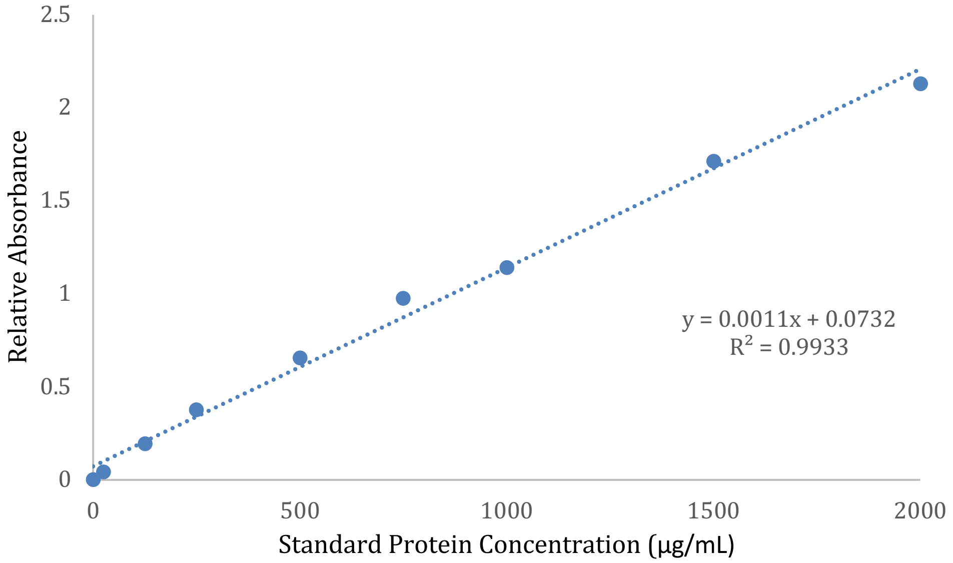

Figure S1. BCA Assay Standard Curve. Protein concentration was measured using a bicinchoninic acid (BCA) protein assay. BCA Abs$^{562\ nm}$ increases linearly with increasing protein concentration; this linear relationship between concentration and absorbance was used to calculate the concentration of *Msmeg* lysates to standardize protein loading for native-PAGE. Standard curve developed using bovine serum albumin (BSA; Bio-rad).

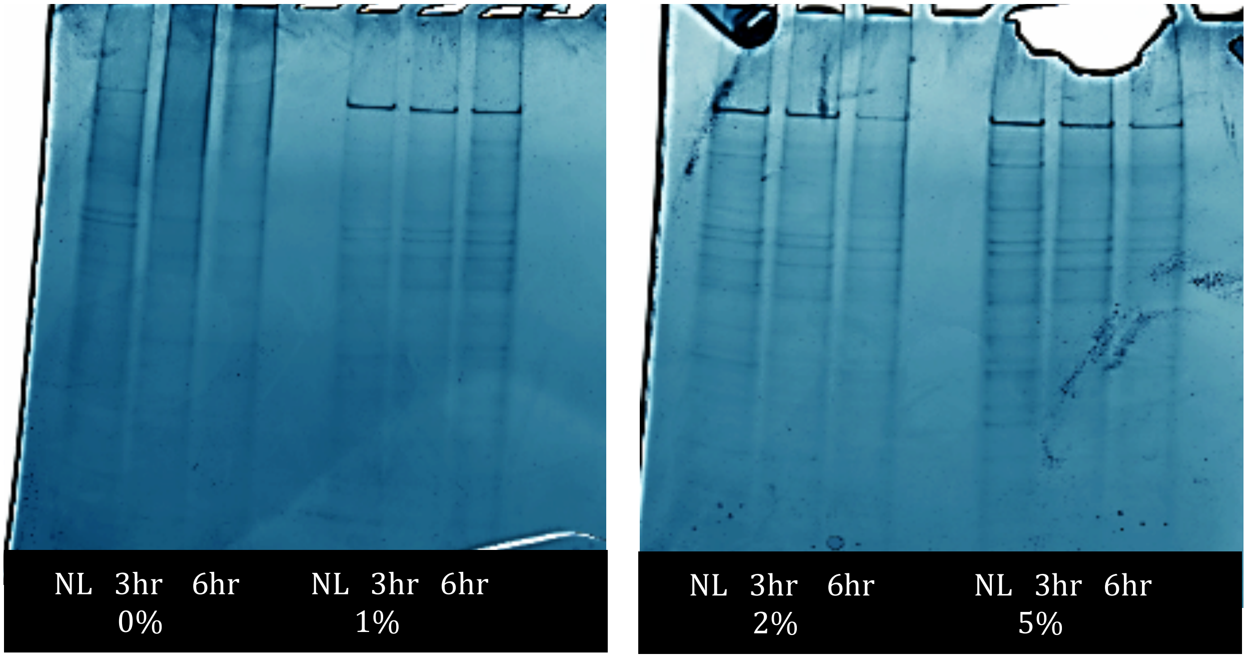

Figure S2. Total Protein Loading native-PAGE samples. *Msmeg* lysates were separated using native-PAGE on a 4-20% polyacrylamide gel. Total protein concentration (12µg) was standardized by BCA assay. Native-PAGE run at 200V for 105 min on ice. Bands were stained with Colloidal Coomassie Blue Stain and visualized using a LI-COR Odyssey® Gel imager.

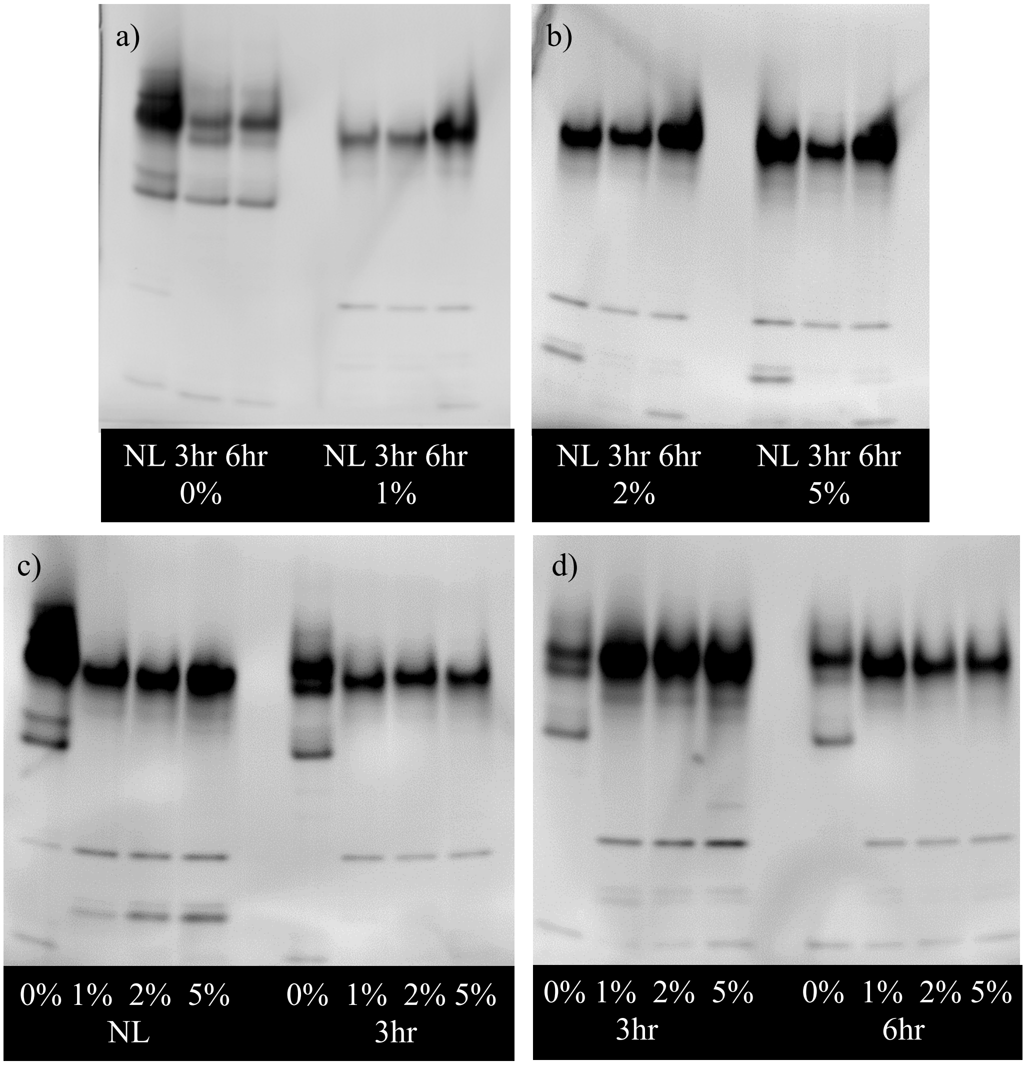

Figure S3. In-gel Enzyme Analysis with Substrate 2. The in-gel activity assay with fluorogenic ester substrate exposure revealed serine hydrolases with differential activity against various substrates across dormant and active growth states (NL=nitrogen limiting, 3hr=3hr activation, 6hr=6hr activation) in various glycerol concentrations (0%, 1%, 2%, 5%). *Msmeg* protein samples were separated using native-PAGE electrophoresis on a 4-20% polyacrylamide gel. The amount of protein in each lane was standardized according to protein concentration as measured with a BCA Assay. After separation, fluorogenic substrate **2** was added to the surface of the gel (5µL of 10 mM in 5mL PBS). After 15 minutes of exposure, LI-COR Odyssey® Gel imaging was used to visualize the present bands. **a** and **b**) correlate serine hydrolase activity changes with increased activation time for each glycerol concentration. **c** and **d)** correlate serine hydrolase activity changes with increased glycerol concentration for each growth state.

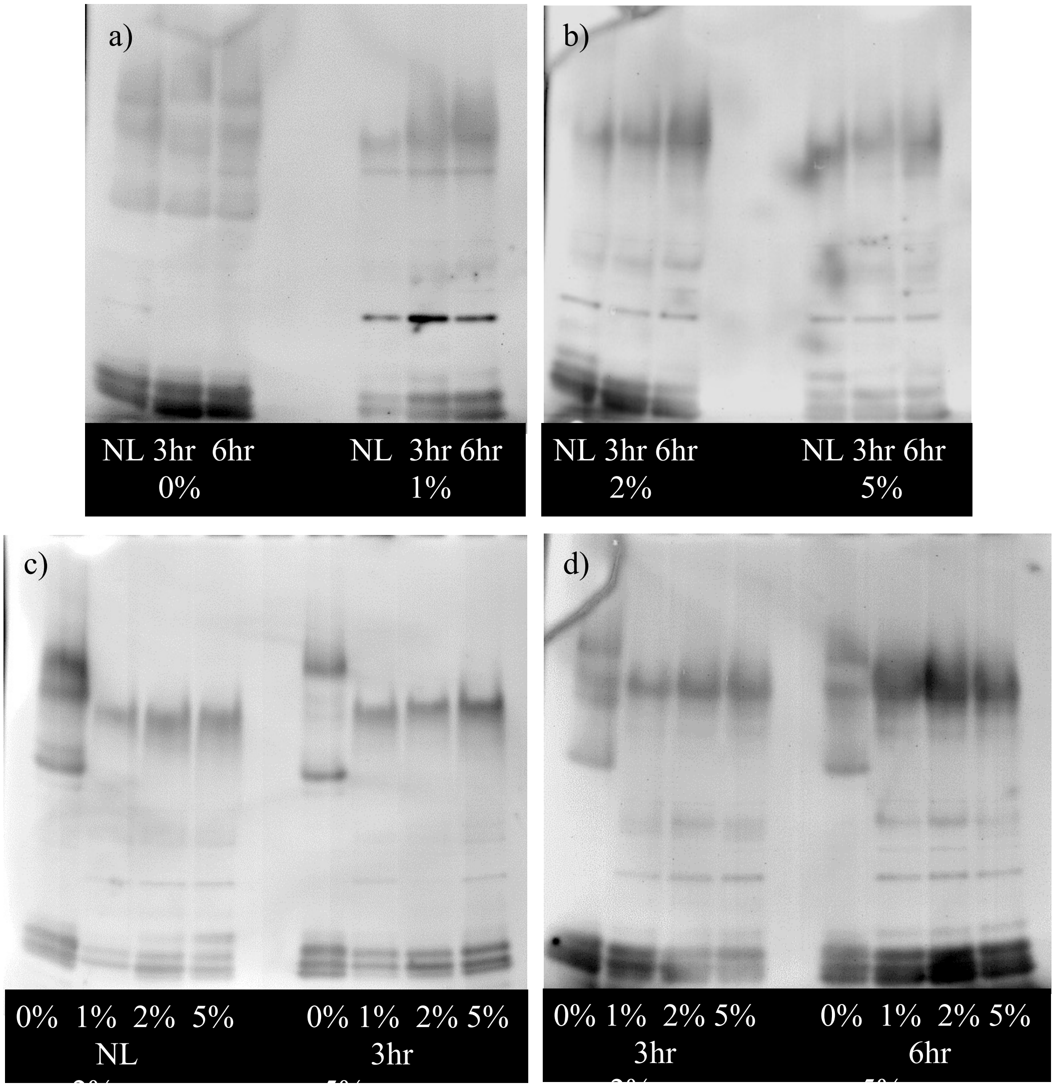

Figure S4. In-gel Enzyme Analysis with Substrate 3. The in-gel activity assay with fluorogenic ester substrate exposure revealed serine hydrolases with differential activity against various substrates across dormant and active growth states (NL=nitrogen limiting, 3hr=3hr activation, 6hr=6hr activation) in various glycerol concentrations (0%, 1%, 2%, 5%). *Msmeg* protein samples were separated using native-PAGE electrophoresis on a 4-20% polyacrylamide gel. The amount of protein in each lane was standardized according to protein concentration as measured with a BCA Assay. After separation, fluorogenic substrate **3** was added to the surface of the gel (5μL of 10 mM in 5mL PBS). After 15 minutes of exposure, LI-COR Odyssey® Gel imaging was used to visualize the present bands. **a** and **b**) correlate serine hydrolase activity changes with increased activation time for each glycerol concentration. **c** and **d)** correlate serine hydrolase activity changes with increased glycerol concentration for each growth state.

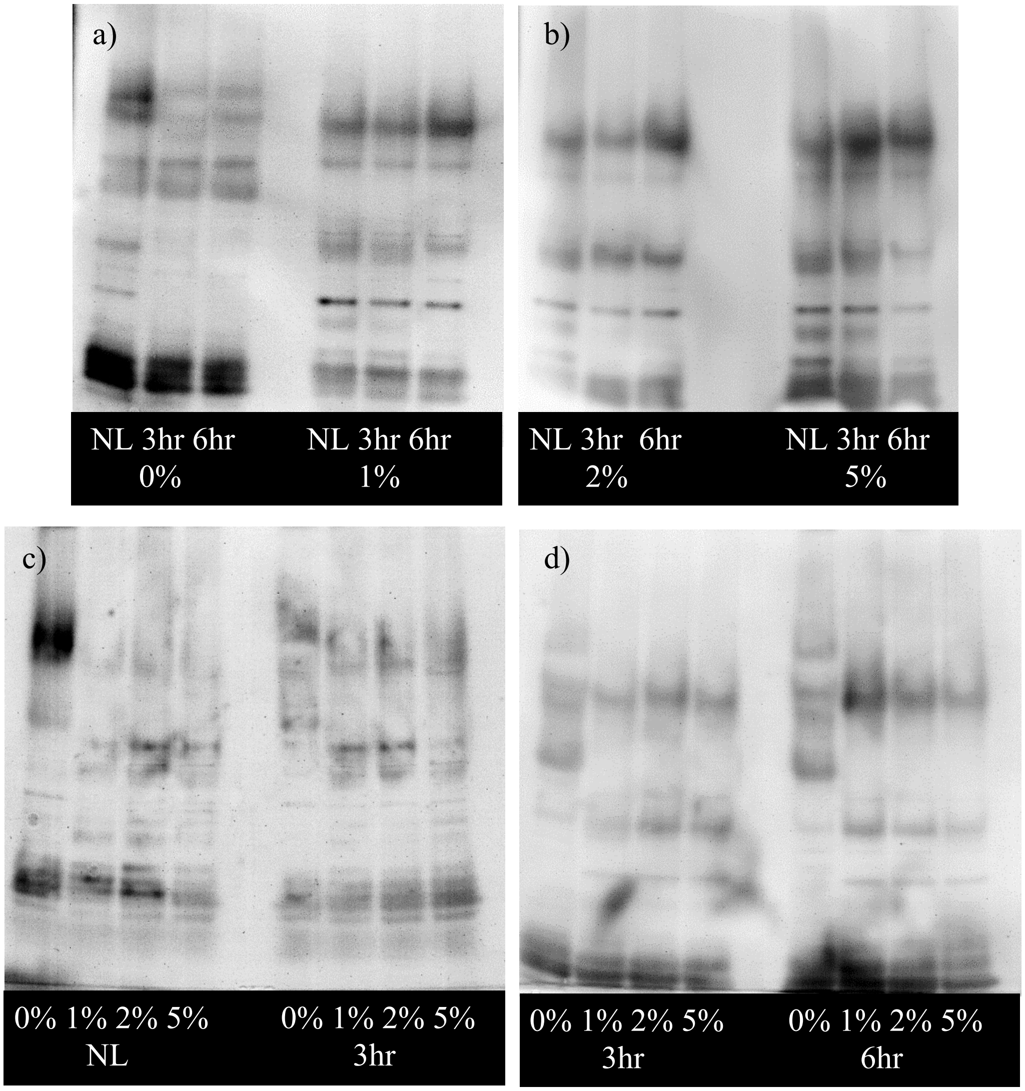

Figure S5. In-gel Enzyme Analysis with Substrate 4. The in-gel activity assay with fluorogenic ester substrate exposure revealed serine hydrolases with differential activity against various substrates across dormant and active growth states (NL=nitrogen limiting, 3hr=3hr activation, 6hr=6hr activation) in various glycerol concentrations (0%, 1%, 2%, 5%). *Msmeg* protein samples were separated using native-PAGE electrophoresis on a 4-20% polyacrylamide gel. The amount of protein in each lane was standardized according to protein concentration as measured with a BCA Assay. After separation, fluorogenic substrate **4** was added to the surface of the gel (5μL of 10 mM in 5mL PBS). After 20 minutes of exposure, LI-COR Odyssey® Gel imaging was used to visualize the present bands. **a** and **b**) correlate serine hydrolase activity changes with increased activation time for each glycerol concentration. **c** and **d)** correlate serine hydrolase activity changes with increased glycerol concentration for each growth state.

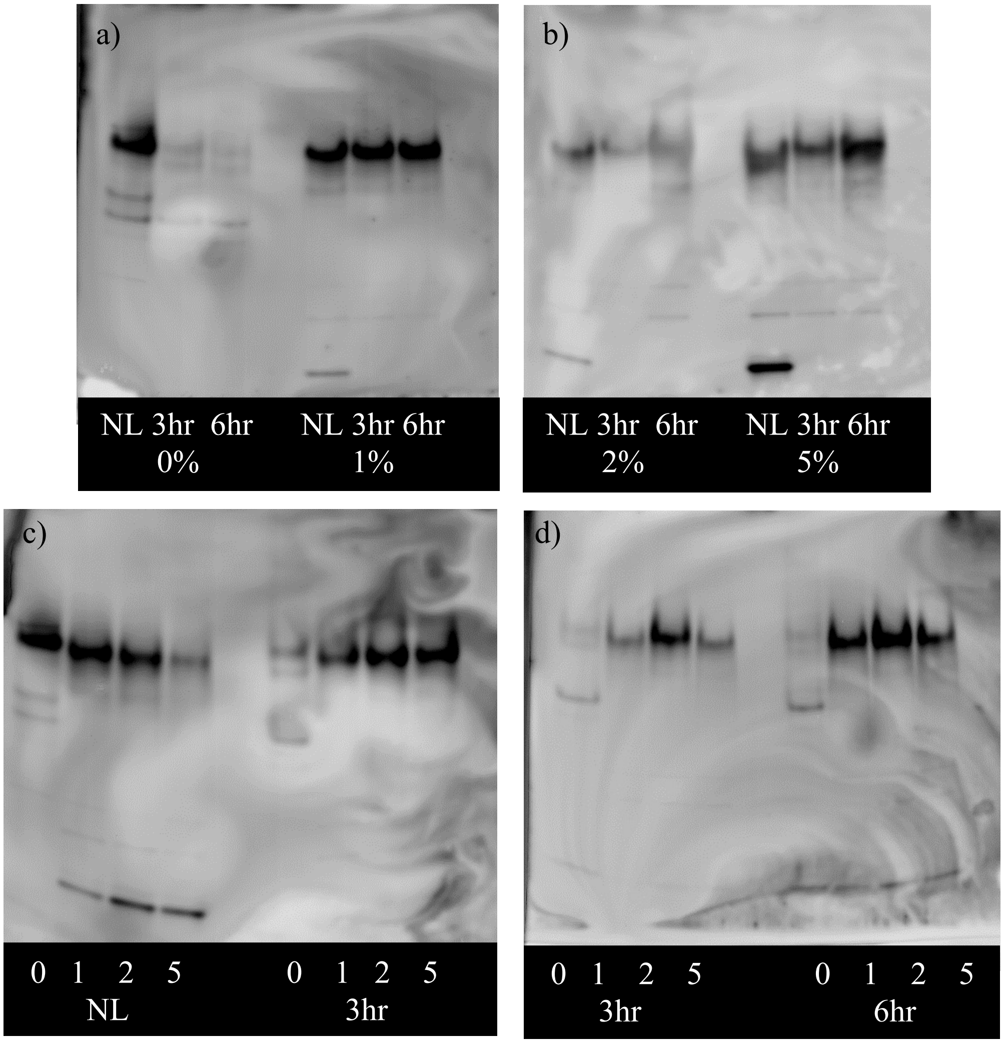

Figure S6. In-gel Enzyme Analysis with Substrate 5. The in-gel activity assay with fluorogenic ester substrate exposure revealed serine hydrolases with differential activity against various substrates across dormant and active growth states (NL=nitrogen limiting, 3hr=3hr activation, 6hr=6hr activation) in various glycerol concentrations (0%, 1%, 2%, 5%). *Msmeg* protein samples were separated using native-PAGE electrophoresis on a 4-20% polyacrylamide gel. The amount of protein in each lane was standardized according to protein concentration as measured with a BCA Assay. After separation, fluorogenic substrate **5** was added to the surface of the gel (20µL of 10 mM in 5mL PBS). After 20 minutes of exposure, LI-COR Odyssey® Gel imaging was used to visualize the present bands. **a** and **b**) correlate serine hydrolase activity changes with increased activation time for each glycerol concentration. **c** and **d)** correlate serine hydrolase activity changes with increased glycerol concentration for each growth state.

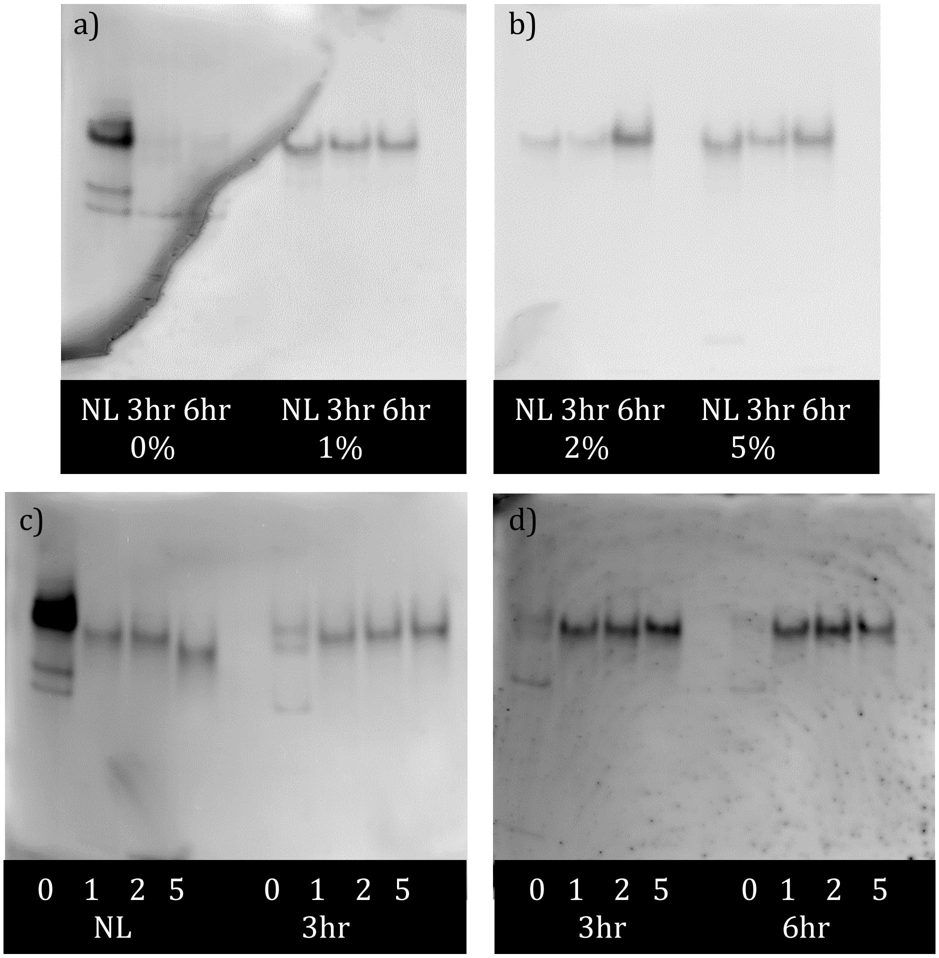

Figure S7. In-gel Enzyme Analysis with Substrate 6. The in-gel activity assay with fluorogenic ester substrate exposure revealed serine hydrolases with differential activity against various substrates across dormant and active growth states (NL=nitrogen limiting, 3hr=3hr activation, 6hr=6hr activation) in various glycerol concentrations (0%, 1%, 2%, 5%). *Msmeg* protein samples were separated using native-PAGE electrophoresis on a 4-20% polyacrylamide gel. The amount of protein in each lane was standardized according to protein concentration as measured with a BCA Assay. After separation, fluorogenic substrate **6** was added to the surface of the gel (20μL of 10 mM in 5mL PBS). After 20 minutes of exposure, LI-COR Odyssey® Gel imaging was used to visualize the present bands. **a** and **b**) correlate serine hydrolase activity changes with increased activation time for each glycerol concentration. **c** and **d)** correlate serine hydrolase activity changes with increased glycerol concentration for each growth state.

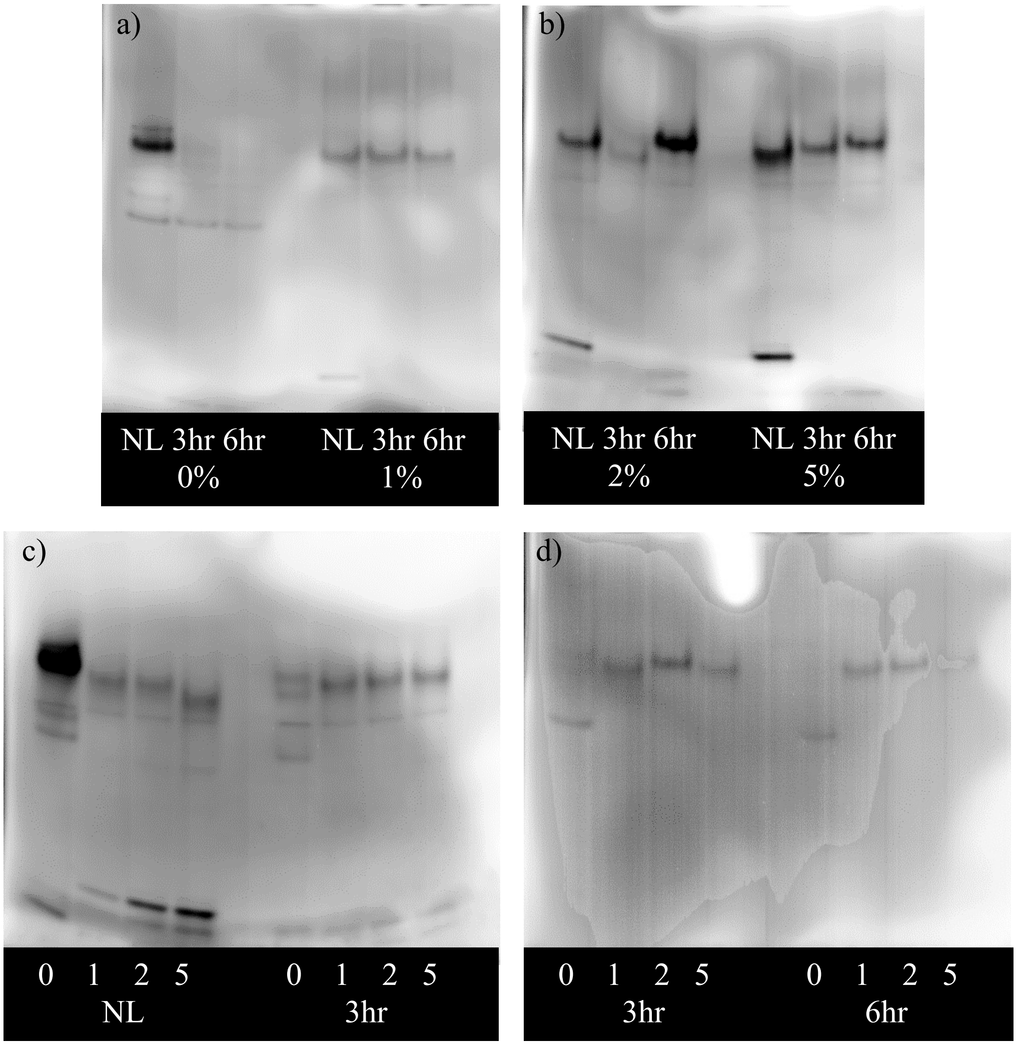

Figure S8. In-gel Enzyme Analysis with Substrate 7. The in-gel activity assay with fluorogenic ester substrate exposure revealed serine hydrolases with differential activity against various substrates across dormant and active growth states (NL=nitrogen limiting, 3hr=3hr activation, 6hr=6hr activation) in various glycerol concentrations (0%, 1%, 2%, 5%). *Msmeg* protein samples were separated using native-PAGE electrophoresis on a 4-20% polyacrylamide gel. The amount of protein in each lane was standardized according to protein concentration as measured with a BCA Assay. After separation, fluorogenic substrate **7** was added to the surface of the gel (20μL of 10 mM in 5mL PBS). After 20 minutes of exposure, LI-COR Odyssey® Gel imaging was used to visualize the present bands. **a** and **b**) correlate serine hydrolase activity changes with increased activation time for each glycerol concentration. **c** and **d)** correlate serine hydrolase activity changes with increased glycerol concentration for each growth state.

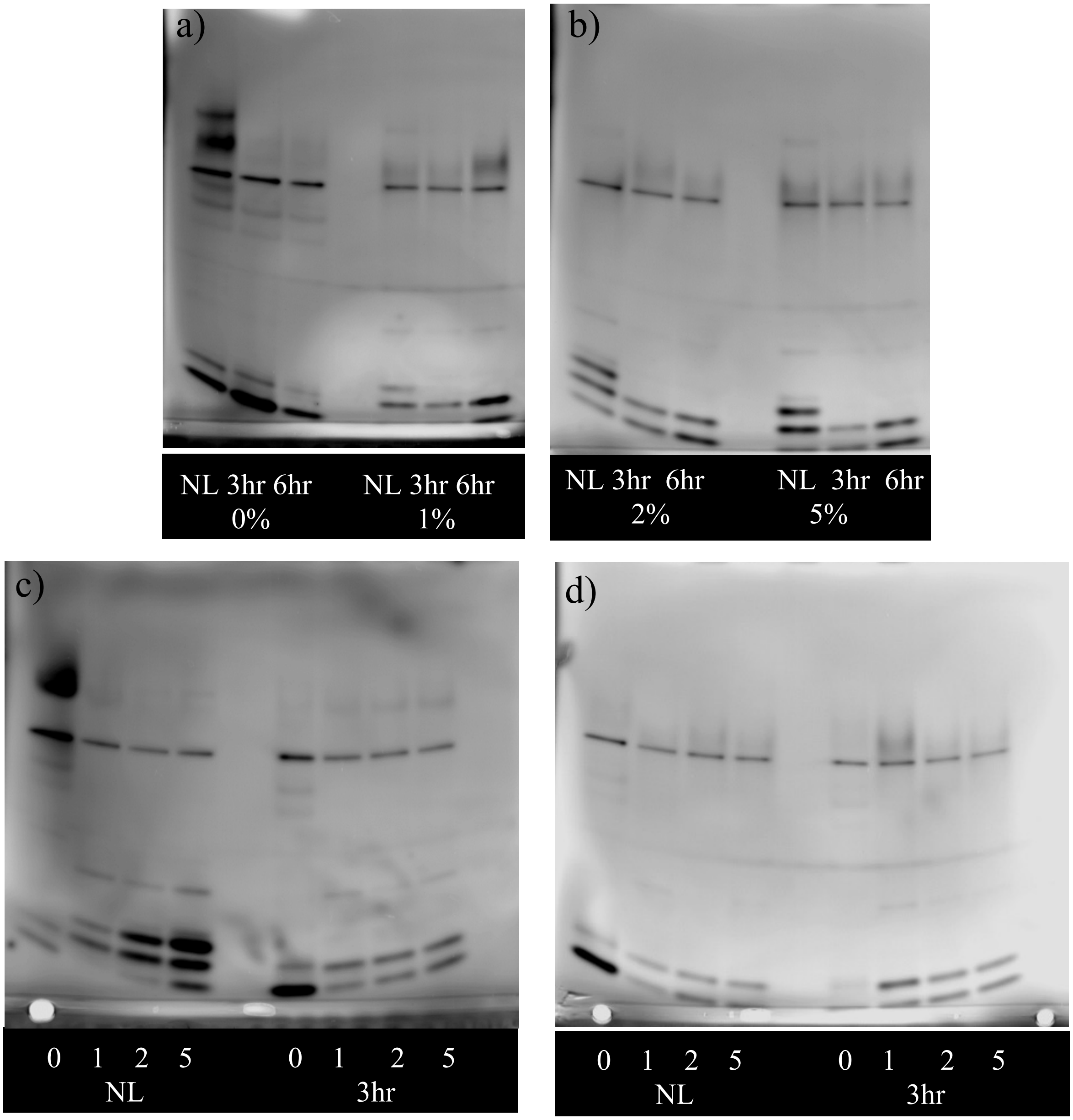

Figure S9. In-gel Enzyme Analysis with Substrate 8. The in-gel activity assay with fluorogenic ester substrate exposure revealed serine hydrolases with differential activity against various substrates across dormant and active growth states (NL=nitrogen limiting, 3hr=3hr activation, 6hr=6hr activation) in various glycerol concentrations (0%, 1%, 2%, 5%). *Msmeg* protein samples were separated using native-PAGE electrophoresis on a 4-20% polyacrylamide gel. The amount of protein in each lane was standardized according to protein concentration as measured with a BCA Assay. After separation, fluorogenic substrate **8** was added to the surface of the gel (20µL of 10 mM in 5mL PBS). After 30 minutes of exposure, LI-COR Odyssey® Gel imaging was used to visualize the present bands. **a** and **b**) correlate serine hydrolase activity changes with increased activation time for each glycerol concentration. **c** and **d)** correlates serine hydrolase activity changes with increased glycerol concentration for each growth state

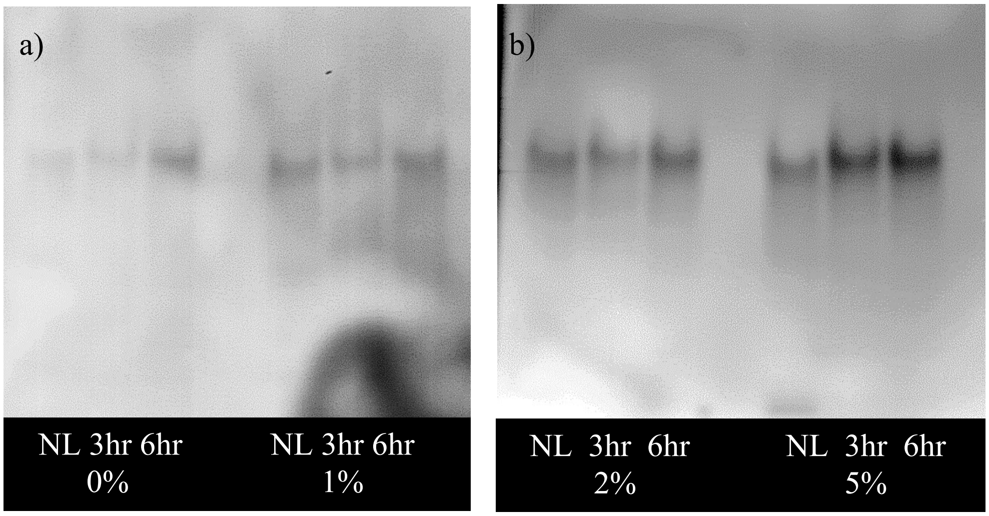

Figure S10. In-gel Enzyme Analysis with Substrates 9 and 10. *Msmeg* protein samples grown in 2% and 5% across various growth states (NL=nitrogen limiting, 3hr=3hr activation, 6hr=6hr activation) were separated using native-PAGE electrophoresis on a 4-20% polyacrylamide gel. The amount of protein in each lane was standardized according to protein concentration as measured with a BCA Assay.[12] After separation, a fluorogenic substrate was added to the surface of the gel (10μL of 10 mM in 5mL PBS). After 30 minutes of exposure, LI-COR Odyssey® Gel imaging was used to visualize the present bands.[9] a) exposure with substrate **9**. b) exposure with substrate **10**.

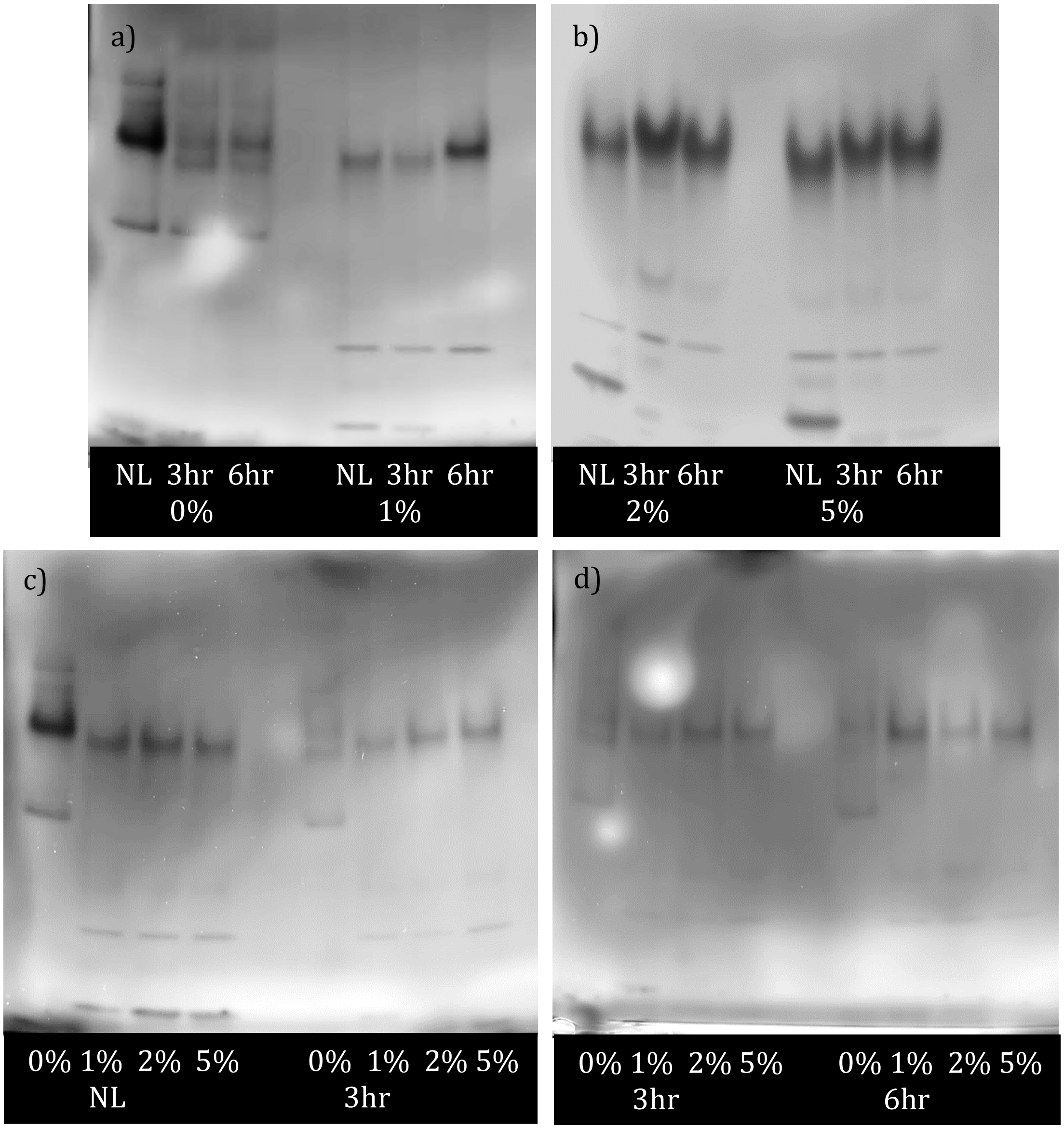

Figure S11. In-gel Enzyme Analysis with Substrate 12. The in-gel activity assay with fluorogenic ester substrate exposure revealed serine hydrolases with differential activity against various substrates across dormant and active growth states (NL=nitrogen limiting, 3hr=3hr activation, 6hr=6hr activation) in various glycerol concentrations (0%, 1%, 2%, 5%). *Msmeg* protein samples were separated using native-PAGE electrophoresis on a 4-20% polyacrylamide gel. The amount of protein in each lane was standardized according to protein concentration as measured with a BCA Assay. After separation, fluorogenic substrate **12** was added to the surface of the gel (20µL of 10 mM in 5mL PBS). After 25 minutes of exposure, LI-COR Odyssey® Gel imaging was used to visualize the present bands. **a** and **b**) correlate serine hydrolase activity changes with increased activation time for each glycerol concentration. **c** and **d)** correlate serine hydrolase activity changes with increased glycerol concentration for each growth state.

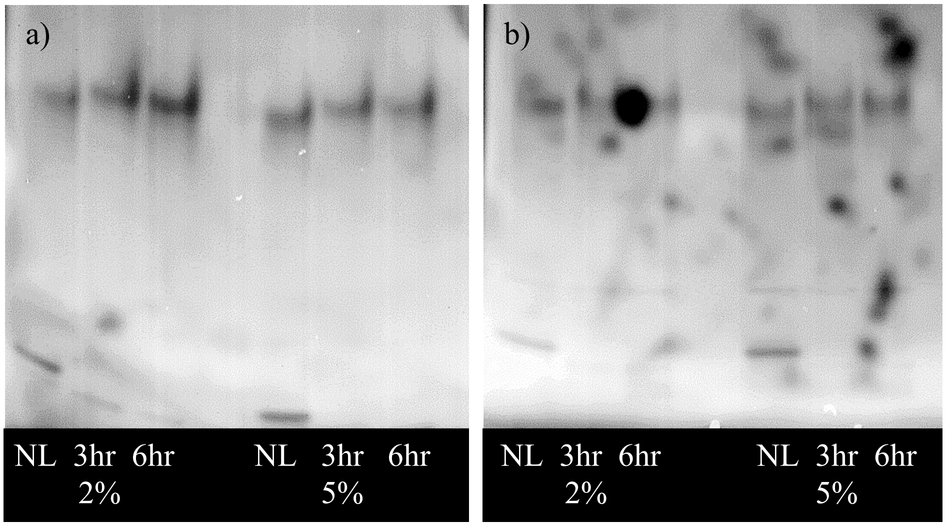

Figure S12. In-gel Enzyme Analysis with Substrates 11 and 13. *Msmeg* protein samples grown in 2% and 5% across various growth states (NL=nitrogen limiting, 3hr=3hr activation, 6hr=6hr activation) were separated using native-PAGE electrophoresis on a 4-20% polyacrylamide gel. The amount of protein in each lane was standardized according to protein concentration as measured with a BCA Assay.[12] After separation, a fluorogenic substrate was added to the surface of the gel (20µL of 10 mM in 5mL PBS). After 25 minutes of exposure, LI-COR Odyssey® Gel imaging was used to visualize the present bands.[9] a) exposure with substrate **11**. b) exposure with substrate **13**.

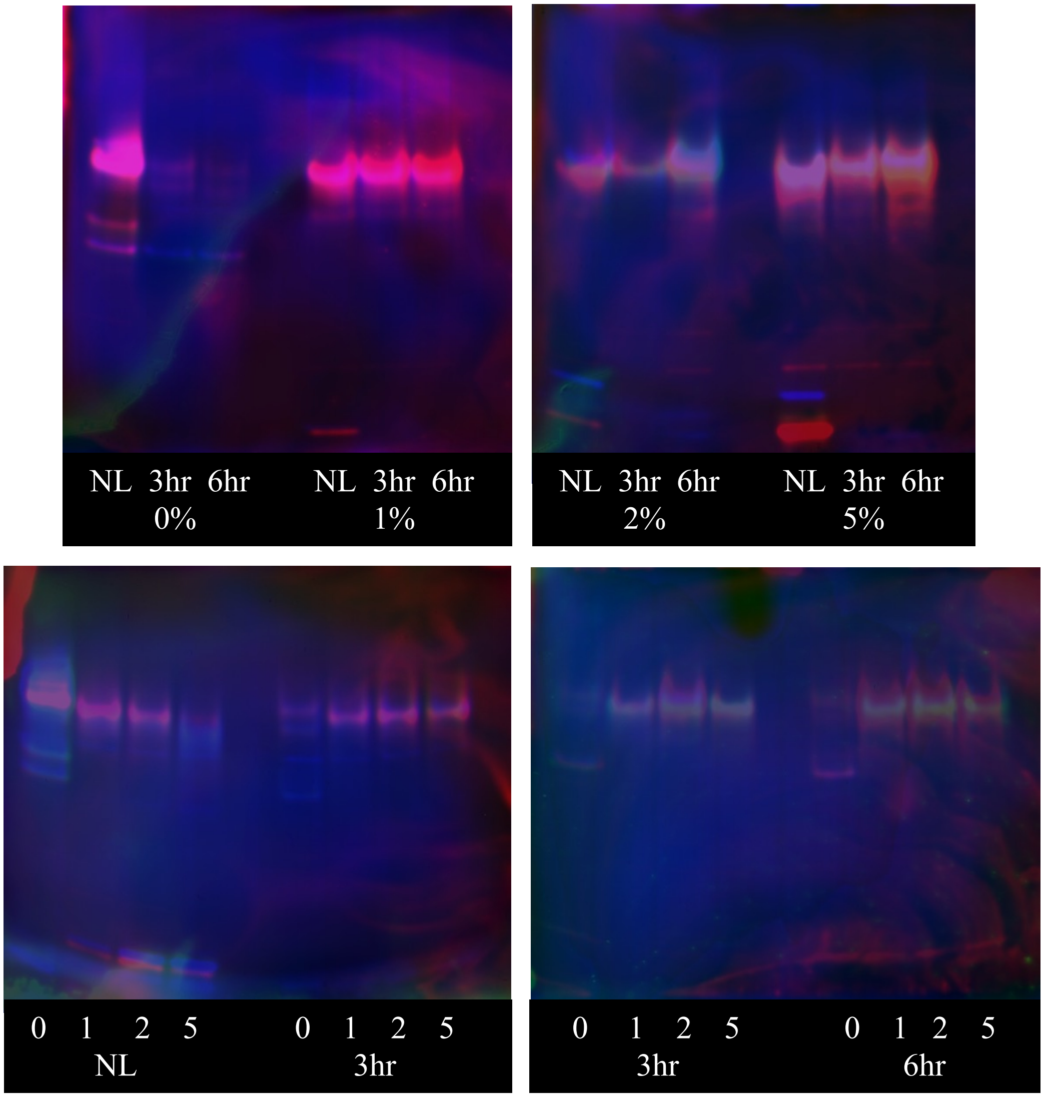

Figure S13. Overlayed In-Gel Analysis of Substrates 5, 6, 7. Images collected for in-gel analysis with fluorogenic substrates **5** (red), **6** (green), and **7** (blue) were overlayed using Adobe Photoshop to reveal serine hydrolases with substrate specificity and those that can be active against multiple substrates. **a** and **b**) correlate serine hydrolase activity changes with increased activation time for each glycerol concentration. **c** and **d**) correlate serine hydrolase activity changes with increased glycerol concentration for each growth state. Individual substrate analysis presented in Figures S6 – S8.

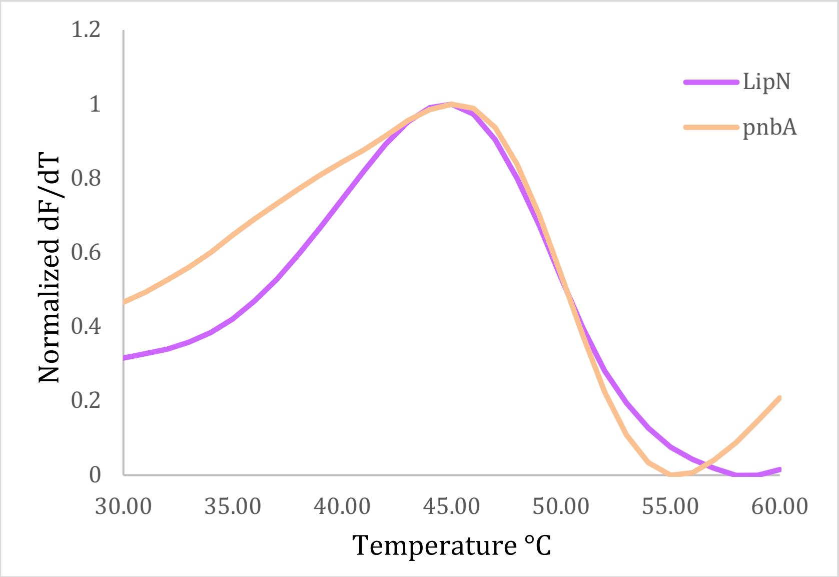

Figure S14. LipN and pnbA Melting Curve. The melting temperatures of LipN (T_M = 46.67 ±0.58°C) and pnbA (T_M = 43.67 ± 2 .52°C) were determined by measuring the increase in Sypro Orange fluorescence (Excitation = 450 – 490 nm, Emission = 610 – 650 nm), as the sample (0.6 mg/mL in PBS) was heated from 15 – 95 °C. The normalized first derivative of the thermal transitions for three independent replicates for both LipN and pnbA are shown.

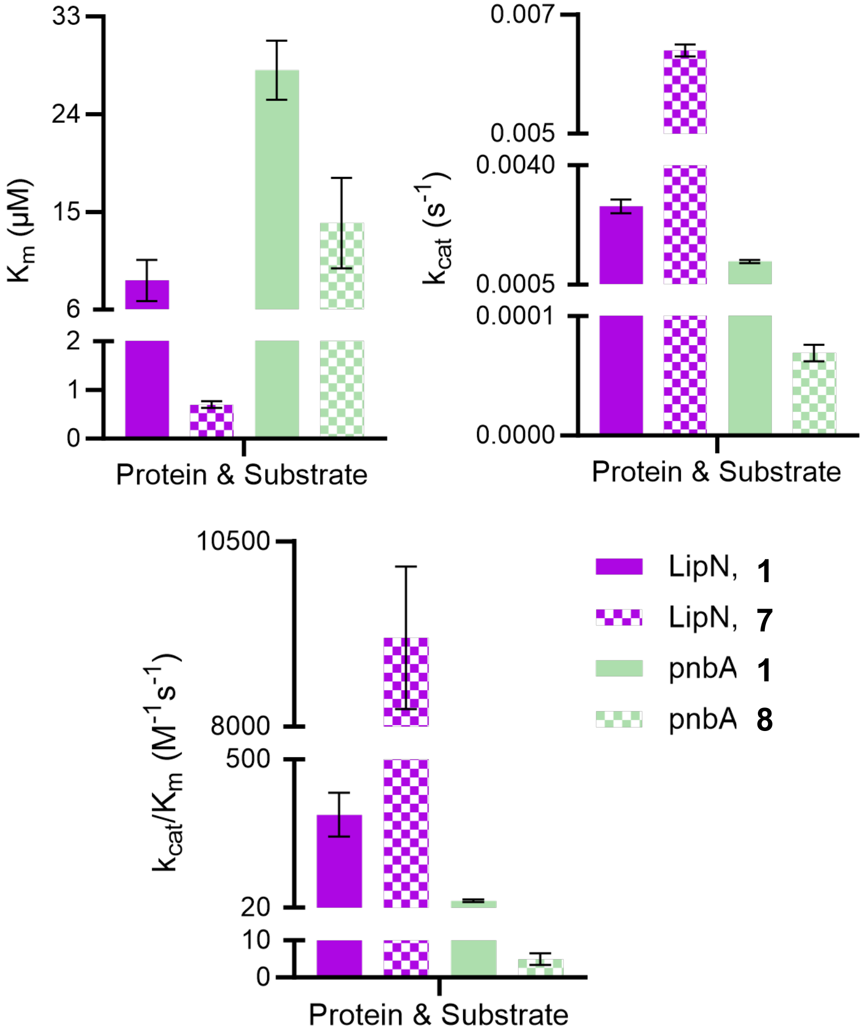

Figure S15. Michaelis Menten Parameters for LipN and pnbA. Enzymatic parameters including the Michaelis constant (K_m), rate constant (k_{cat}), and catalytic efficiency (k_{cat}/K_m), for LipN and pnbA against various substrates were calculated (n=3) using Graphpad Prism.

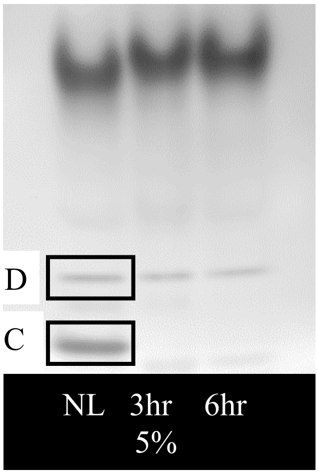

Band	MS Protein Hits	Protein Function	*Msmeg* gene	*Mtb* analog
C	α/β hydrolase	Lipase/esterase activity	MSMEG_3842	lipV
	CocE hydrolase	Cocaine esterase; α/β fold	CocE_1	
	Polyketide synthetase	Lipid metabolism & fatty acid biosynthesis	MSMEG_4512	pks5
	Patatin-like protein	Lipid storage protein with lipase activity	MSMEI_5589	
D	α/β hydrolase	Lipase/esterase activity	MSMEG_3842	lipV
	CocE hydrolase	Cocaine esterase; α/β fold	CocE_1	
	Fatty acid CoA synthase	In metabolic pathway; catalyzes FA-CoA formation	MSMEG_5970	argD, hemL

Figure S16. Potential Serine Hydrolase Identities for Two Hydrolase Bands Determined by Mass Spectrometry. *Msmeg* proteins were separated and exposed to fluorogenic ester substrate **12.** a) In-gel activity assay results. The serine hydrolase present in band C is present only in NL conditions. The serine hydrolase present in band D is active at a constant level across all growth states. These two bands were narrowly excised from the native-PAGE and sent for MALDI MS-MS peptide fingerprinting protein identification by Applied Biomics. Mascot results for the top ten hits for available peptide fragments were provided and the identity of potential proteins with serine hydrolase activity were further analyzed in part b. b) The excision of bands C and D and identification by MS revealed potential protein hits when checked against various protein databases. These potential identities, the biological processes they are involved in, and the genes responsible are shown above.
